# Longitudinal stability in cigarette smokers of urinary eicosanoid biomarkers of oxidative damage and inflammation

**DOI:** 10.1371/journal.pone.0215853

**Published:** 2019-04-25

**Authors:** Steven G. Carmella, Alisa K. Heskin, Mei Kuen Tang, Joni Jensen, Xianghua Luo, Chap T. Le, Sharon E. Murphy, Neal L. Benowitz, F. Joseph McClernon, Ryan Vandrey, Sharon S. Allen, Rachel Denlinger-Apte, Paul M. Cinciripini, Andrew A. Strasser, Mustafa al’Absi, Jason D. Robinson, Eric C. Donny, Dorothy K. Hatsukami, Stephen S. Hecht

**Affiliations:** 1 Masonic Cancer Center, University of Minnesota, Minneapolis, Minnesota, United States of America; 2 Division of Biostatistics, School of Public Health, University of Minnesota, Minneapolis, Minnesota, United States of America; 3 Department of Medicine, University of California San Francisco, San Francisco, California, United States of America; 4 Department of Psychiatry and Behavioral Sciences, Duke University, Durham, North Carolina, United States of America; 5 Department of Psychiatry and Behavioral Sciences, Johns Hopkins University, Baltimore, Maryland, United States of America; 6 Department of Family Medicine and Community Health, University of Minnesota Medical School, Minneapolis, Minnesota, United States of America; 7 Department of Behavioral and Social Sciences, Brown University, Providence, Rhode Island, United States of America; 8 Department of Behavioral Science, The University of Texas MD Anderson Cancer Center, Houston, Texas, United States of America; 9 Department of Psychiatry, University of Pennsylvania, Philadelphia, Pennsylvania, United States of America; 10 Behavioral Medicine Laboratories, University of Minnesota Medical School, Duluth, Minnesota, United States of America; 11 Department of Physiology and Pharmacology, Wake Forest School of Medicine, Winston-Salem, North Carolina, United States of America; National Yang-Ming University, TAIWAN

## Abstract

The urinary metabolites (*Z*)-7-[1*R*,2*R*,3*R*,5*S*)-3,5-dihydroxy-2-[(*E*,3*S*)-3-hydroxyoct-1-enyl]cyclopentyl]hept-5-enoic acid (8-*iso*-PGF_2α_), an F2-isoprostane and biomarker of oxidative damage, and “prostaglandin E_2_ metabolite” (PGE-M), a biomarker of inflammation, are elevated in cigarette smokers. However, there is little information in the literature on the longitudinal stability of these widely used biomarkers. In a large clinical trial involving 10 institutional sites, smokers were given, free of charge over a period of 20 weeks, Spectrum NRC600/601 research cigarettes containing 15.5 mg nicotine/g tobacco. All participants were instructed to smoke these cigarettes for the duration of the study. At weeks 4, 8, 12, 16, and 20, first morning urine voids were collected and analyzed for 8-*iso*-PGF_2α_ and PGE-M using validated liquid chromatography-electrospray ionization-tandem mass spectrometry methods. The mean level of 8-*iso*-PGF_2α_ at Week 4 was 1.34 ± 1.08 (S.D.) pmol/mg creatinine (N = 226) while that of PGE-M was 73.7 ± 113 (S.D.) pmol/mg creatinine (N = 232). The corresponding levels at Week 20 were 1.35 ± 0.93 (S.D.) pmol/mg creatinine (N = 209) for 8-*iso*-PGF_2α_ and 74.2 ± 142 (S.D.) pmol/mg creatinine (N = 210) for PGE-M. There was variation in these values in the intervening weeks. The intra-class correlation coefficients (ICC) were 0.51 (95% CI, 0.45, 0.57) and 0.36 (0.30, 0.43), for 8-*iso*-PGF_2α_ and PGE-M, respectively, indicating fair longitudinal stability for 8-*iso*-PGF_2α_ and poorer longitudinal stability for PGE-M in cigarette smokers. Males had higher ICC values than females for both 8-*iso*-PGF_2α_ and PGE-M. These results indicate that, in addition to cigarette smoking, endogenous processes of oxidative damage and inflammation influence the levels of these biomarkers over time among current smokers.

## Introduction

**Fig 1:**
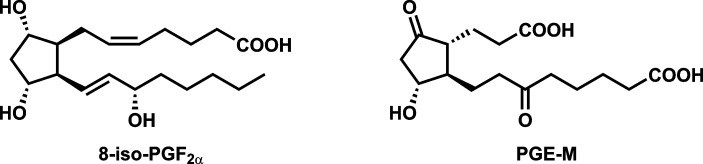
Structures of 8-*iso*-PGF_2α_ and PGE-M.

The closely linked phenomena of oxidative damage and inflammation play a significant role in diseases caused by cigarette smoking including cancer, cardiovascular disease, and chronic obstructive pulmonary disease [1, 2]. As an example, extensive research has clearly demonstrated that cigarette smoke and its condensate have tumor promoting and co-carcinogenic activity, properties that are associated with oxidative damage and inflammation and are critical in cancer induction [1, 2]. As noted below, the urinary metabolites (*Z*)-7-[1*R*,2*R*,3*R*,5*S*)-3,5-dihydroxy-2-[(*E*,3*S*)-3-hydroxyoct-1-enyl]cyclopentyl]hept-5-enoic acid (8-*iso*-PGF_2α_), an F2-isoprostane and biomarker of oxidative damage, and “prostaglandin E_2_ metabolite” (PGE-M)(**Fig 1**), a biomarker of inflammation, are elevated in smokers. Furthermore, a recent nested case-control study within the prospective Shanghai Cohort Study demonstrated a significant relationship of 8-*iso*-PGF_2α_ to lung cancer incidence in cigarette smokers [3]. Thus, 8-*iso*-PGF_2α_ and PGE-M are considered “biomarkers of potential harm” reflecting early biological effects strongly associated with disease [4].

Morrow *et al* were the first to demonstrate increased levels of F2-isoprostanes in the urine of smokers compared to non-smokers [5]. Since then, multiple studies have reliably shown that smokers have increased 8-*iso*-PGF_2α_ in urine compared to non-smokers [6–12]. Greater intensity of smoking behavior (e.g. more cigarettes per day) is associated with higher urinary levels of 8-*iso*-PGF_2α_ [13, 14]. Studies have also shown that levels of 8-*iso*-PGF_2α_ decrease upon smoking cessation [13, 15, 16]. Together, these data clearly show that cigarette smoking increases levels of 8-*iso*-PGF_2α_, indicating oxidative damage.

The Dannenberg group was the first to observe higher levels of PGE-M in ever smokers compared to never smokers and in current versus former smokers [17]. Okayasu *et al* developed an immunoassay for PGE-M and found significantly higher levels in smokers than in never smokers [18]. Mohebati *et al* showed that a combination of zileuton, a 5-lipoxygenase inhibitor, and celecoxib, a selective COX-2 inhibitor, decreased levels of PGE-M in smokers [19]. Kekatpure *et al* demonstrated that urinary PGE-M levels were increased in healthy tobacco quid chewers compared with never tobacco quid chewers-never smokers [20].

To our knowledge, there is only one report in the literature that has repeatedly examined levels of 8-*iso*-PGF_2α_ and PGE-M over an extended period of time. That study was carried out in subjects (56% smokers) from the Shanghai Men’s Health Study [21]. There are no reports in the literature on the longitudinal stability of urinary levels of 8-*iso*-PGF_2α_ and PGE-M, exclusively in cigarette smokers. Our recent randomized clinical study that compared the impact of gradually versus immediately reducing cigarette nicotine content to very low levels included a control group that was given Spectrum research cigarettes containing 15.5 mg nicotine per gram tobacco (a level consistent with currently marketed cigarettes), free of charge, for a period of 20 weeks [22]. Measurements of biomarkers at 5 time points in this group provided an ideal opportunity to assess the quantitative stability of these biomarkers within the same individuals, using the same cigarettes, over an extended period of time. It is important to know the variation in a biomarker over time under relatively constant conditions in order to properly assess the effects of changes in tobacco use on their levels. This is particularly true for biomarkers of potential harm such as 8-*iso*-PGF_2α_ and PGE-M, which decrease only modestly upon smoking cessation.

## Materials and methods

### Clinical study

The control group of a randomized clinical trial (clinicaltrials.gov identifier NCT02139930) involving 10 institutional sites is the source of the data reported here. The trial determined the effect of immediate vs. gradual reduction in nicotine content of cigarettes on biomarkers of smoke exposure. Details of this trial have been reported [22]. The study protocol was approved by the University of Minnesota Institutional Review Board (IRB Code Number 1106M01561, FWA Number 00000312). Written consent was obtained from all participants. Briefly, the present study included participants in the control group, consisting of cigarette smokers randomly assigned to smoke Spectrum NRC600/601 research cigarettes, containing 15.5 mg nicotine/g tobacco. Participants received the research cigarettes (either menthol or non-menthol according to their preference) free of charge over a period of 20 weeks. Participants attended a weekly clinic visit for the first 4 weeks and then bi-weekly visits for the next 16 weeks. At each clinic visit, participants received twice the number of cigarettes reported at baseline. At all visits, smokers were advised on the importance of smoking only the study cigarettes, as long as they continued to smoke. They were asked to return full, partial or empty cigarette packs and the extent of concordance of self-reported cigarette consumption and packs returned were determined. If they were widely discrepant, an inquiry was made regarding the discrepancy. Bonus payments were provided for those who were considered compliant. Support was given for attempts to quit smoking if interest were expressed. At weeks 4, 8, 12, 16, and 20, first morning urine voids were collected for measurement of biomarkers.

### Analysis of 8-*iso*-PGF_2α_ in urine

The method was based on that described by Yan *et al* [7]. 8-*iso*-PGF_2α_ and 8-*iso*-PGF_2α_-d_4_ were procured from Cayman Chemical (Ann Arbor, MI). For each sample, a 200 μL aliquot of urine was transferred to a well in a V-bottom 96-well collection plate (Analytical Sales and Services, Cat. # 59623–23). Twenty μL of 0.1 ng/μL 8-*iso*-PGF_2α_-d_4_ dissolved in 80% aqueous methanol and 20 μL of formic acid (ACS grade, ≥98%) were added to each sample. The plate was covered with a 96-square well sealing mat (Phenomenex, Cat. # AH0-8597) and the samples were vortexed. Each sample plate also contained 3 positive and 2 negative controls in the forms of pooled (n = 9) smokers’ urine and H_2_O, respectively. Bond Elut C18 96 square-well plates (100 mg) from Agilent (Cat. # A396011C) were used for solid phase extraction (SPE). The plates were processed on a CEREX System 96 processor. Each plate was conditioned sequentially with 1 mL of MeOH, 1 mL of CH_3_CN, and 3 mL of 50 mM potassium phosphate buffer, pH 3. Following application of the samples, the plate was washed with additional volumes of phosphate buffer and hexanes, 3 mL each, before elution with 1 mL ethyl acetate into a Tru-Taper 96-well plate (Analytical Sales and Services, Cat. # 968820). The ethyl acetate was evaporated to dryness using a Speedvac concentrator (Thermo Fisher), and the plates were capped and stored at -20°C until LC -electrospray ionization (ESI^-^)- MS/MS analysis. For injection, samples were reconstituted in 30 μL 80:20 H_2_O:MeOH (v/v) with 0.15% NH_4_OH.

Sample analysis was performed using LC ESI^—^MS/MS on a Vantage triple quadrupole mass spectrometer (Thermo Scientific, Pittsburgh PA) with a Dionex Ultimate 3000 Rapid separation (RSLC) HPLC system (Thermo Scientific, Pittsburgh PA). An XBridge BEH C18, 2.5 μm, 50 mm x 1.0 mm column (Waters Corp Cat. # 186003118) was used. Sample injection volumes were 2–5 μL. The column was kept at room temperature and H_2_O and 95:5 CH_3_CN:MeOH (v/v) both containing 0.15% NH_4_OH were the eluting solvents. A shallow elution gradient with a flow rate of 45 μL/min was used to achieve separation of 8-*iso*-PGF_2α_ from its isomers. After the initial condition of 5% organic, the percentage was increased to 12% over 0.5 min, held at 12% for 2 min, increased to 16% over 3 min, and held at 16% for 2 min before increasing to 95% organic over 4 min, holding for 2 min, and re-equilibrating at initial conditions for 2.5 min. Transitions monitored were: 8-*iso*-PGF_2α_, *m/z* 353 → *m/z* 193 (quantifier) and *m/z* 353 → *m/z* 173 (qualifier); 8-*iso*-PGF_2α_-d_4_, *m/z* 357 → *m/z* 197 (quantifier) and *m/z* 357 → *m/z* 177 (qualifier). Fragmentation was achieved with a collision energy of 25 V and gas pressure of 1.2 mTorr. Argon was the collision gas. Quadrupole resolution was set at *m/*z 0.7 for both Q1 and Q3, with a scan width of *m/z* 0.1 and scan time of 0.25 s. The capillary temperature was set to 255°C, N_2_ sheath gas pressure at 40, and N_2_ auxiliary gas pressure at 2 psi.

### Analysis of PGE-M in urine

The PGE-M analysis was performed as described previously with modifications [23]. For each sample, a 400 μL aliquot of urine was transferred to a well in a 2 mL V-bottom 96-well collection plate (Analytical Sales and Services, Cat. # 59623–23) and was spiked with 120 pmol PGEM-d_6_ (Cayman Chemical, Ann Harbor, MI) dissolved in 10% aqueous formic acid and 40 μL of formic acid (ACS grade ≥98%). The plates were capped with a sealing mat (Phenomenex, Cat. # AH0-8597), vortexed and heated for 14–16 h at 60°C to convert PGE-M to PGA-M by dehydration. Each sample plate also contained 3 positive and 2 negative controls as pooled smokers’ (n = 9) urine and H_2_O, respectively. Bond Elut C18 96 square-well plates (100 mg) from Agilent (Cat. # A396011C) were used for solid phase extraction. The plates were processed on a CEREX System 96 processer. Each plate was conditioned sequentially with 1 mL of MeOH, 1 mL of CH_3_CN and 3 mL of 50 mM potassium phosphate buffer, pH 3. Following application of the samples, the plate was washed with additional volumes of phosphate buffer and hexanes, 3 mL each, before elution with 1.25 mL ethyl acetate into a Tru-Taper 96-well collection plate (Analytical Sales and Services, Cat. # 968820). The ethyl acetate was evaporated to dryness using a Speedvac concentrator (Thermo Fisher), and the plates were capped and stored in a -20°C freezer until LC-MS/MS analysis. For injection, samples were reconstituted in 50 μL of 5% aqueous CH_3_CN with 0.1% formic acid.

Ten μL of the reconstituted sample was injected onto the LC-MS/MS system for SRM analysis on a TSQ Quantum Discovery Max instrument (Thermo Fisher Scientific, Waltham, MA) operated in the negative ion APCI mode. Chromatography was performed on an Agilent 1100 HPLC system (Agilent Technologies, Inc., Santa Clara, CA) with a 50 x 3.0 mm, 2.6 μm Kinetex C18 LC column (Phenomenex, Torrance, CA). Mobile phase A was 15 mM ammonium acetate and solvent B was CH_3_OH. The LC column temperature was 45°C and the flow rate was 0.34 mL/min. The column gradient was at 98% A and 2% B for 2 min, then ramped up to 12% B over 3 min and held there for 2 min. Then, solvent B was increased to 95% within 0.5 min and held for 1 min. Finally, the column was re-equilibrated for 4 min at initial conditions. The transitions monitored were the following: PGA-M-d_6_, *m/z* 315 → *m/z* 297 (quantifier) and *m/z* 315 → 149 (qualifier); PGA-M, *m/z* 309 → *m/z* 291 (quantifier) and *m/z* 309 → 143 (qualifier). Other MS/MS parameters were as follows: collision energy for transitions; 15 V for *m/z* 315 → *m/z* 297, *m/z* 309 → *m/z* 291 and 23 V for *m/z* 315 → *m/z* 149, *m/z* 309 → *m/z* 143; Q1 = 0.7 FWHM and Q3 = 0.7 FWHM; scan width *m/z* = 0.10; scan time 0.25 s; vaporizer temperature 80°C; capillary temperature 270°C; N_2_ sheath gas pressure, 60 psi; N_2_ auxiliary gas pressure, 5 psi. Argon was the collision gas.

### Preparation of pooled smokers’ urine samples

The pooled smokers’ urine reference material was prepared by combining the 24h voids from nine smokers. The smoking status was confirmed by analysis of total cotinine which averaged 1785 ng/ml. The self-reported cigarettes per day averaged 16.3. The non-smoker reference material was prepared by combining the 24h voids from six non-smokers.

### Analysis of total nicotine equivalents (TNE)

TNE, the sum of nicotine, cotinine, 3’-hydroxycotinine and their glucuronides and nicotine *N*-oxide, were analyzed by liquid chromatography-tandem mass spectrometry using stable isotope labelled internal standards as described previously [24].

### Analysis of urinary creatinine

Creatinine was analyzed using a colorimetric microplate assay (CRE34-K01) purchased from Eagle Bioscience as described (https://eaglebio.com/product/creatinine-microplate-assay-kit/).

### Statistical methods

Following Hankinson *et al*, [25] the intraclass correlation coefficients (ICC) and 95% confidence intervals (CI) were estimated using the SAS macro %ICC9 [26], where a linear mixed model for log-transformed biomarkers was used with a random intercept. The ICC was estimated for both the overall group and for groups defined by gender, body mass index (BMI <25, 25–29, ≥ 30), age (<40, ≥ 40), and amount of smoking at randomization (<10 cigarettes per day, ≥ 10 cigarettes per day). The coefficient of variation (CV) for each control participant who had ≥ 2 measurements was calculated as the sample standard deviation divided by the sample mean. The CV of each biomarker was regressed on gender, BMI, age and amount of smoking at randomization using bivariate linear regression, where BMI, age, and amount of smoking were continuous.

Gender, BMI, age, and amount of smoking at randomization were analyzed in terms of their association with the levels of the biomarkers. Summary statistics including mean, median, and interquartile ranges of the biomarkers are presented for weeks 4–20 using boxplots. The repeated measures of log-transformed biomarkers were analyzed for each factor using a linear mixed model with a fixed effect of the studied factor and a random intercept. The p-value of the fixed effect of the studied factor is reported. Note that for missing data (<10%), the linear mixed models that we used were based on the missing at random (MAR) assumption [27].

## Results

We assessed the biomarkers in the smokers of Spectrum cigarettes as summarized in **Table 1**. The mean age of the smokers was 45.0 ± 13.4 years. They were 61% white, 30% African American, and 9% other races. They smoked a mean of 20.6 ± 11.4 cigarettes per day, 97.7% of which were the Spectrum research cigarettes. Further characteristics of the group have been reported [22]. The methods for 8-*iso*-PGF_2α_ and PGE-M were validated in our laboratory. For 8-*iso*-PGF_2α_, the average value obtained upon analysis of 290 pooled smokers’ urine control samples distributed in 97 plates throughout the course of a recent study was 1.03 ± 0.072 pmol/mL urine giving an overall inter-assay precision (%CV) of 7.0%. The intra-assay precision from the assay of 8 replicates was 2.9%. To assess accuracy, three levels of 8-*iso*-PGF_2α_ were added in triplicate to samples of pooled smokers’ urine and assayed. The added levels were 0.564, 1.41 and 14.1 pmol/mL. This was repeated on two additional days. The average inter-assay accuracies for each level were 104%, 98% and 102% respectively. The calculated assay limit of detection (LOD) was 0.03 pmol/mL.

**Table 1:** Means and other summary statistics for 8-*iso*PGF_2α_ and PGE-M per mg creatinine at each week of the study in the subjects who smoked the Spectrum cigarettes.

Week	Variable	N	Mean	Std Dev	Std Error	Min	Max	Median	Geometric Mean
4	8-*iso*PGF_2α_/creatinine (pmol/mg) PGE-M/creatinine (pmol/mg)	226 232	1.34 73.7	1.08 113	0.07 7.39	0.08 0.36	13.5 1520	1.12 46.2	1.14 49.0
8	8-*iso*PGF_2α_/creatinine (pmol/mg) PGE-M/creatinine (pmol/mg)	215 222	1.32 74.8	0.83 88.5	0.06 5.94	0.02 0.49	6.72 788	1.11 50.9	1.12 49.2
12	8-*iso*PGF_2α_/creatinine (pmol/mg) PGE-M/creatinine (pmol/mg)	210 217	1.28 75.4	0.74 121	0.05 8.22	0.12 0.95	4.976 1550	1.06 47.2	1.10 48.3
16	8-*iso*PGF_2α_/creatinine (pmol/mg) PGE-M/creatinine (pmol/mg)	206 212	1.26 80.7	0.73 92.2	0.05 6.34	0.18 0.63	4.72 906	1.05 54.9	1.10 54.8
20	8-*iso*PGF_2α_/creatinine (pmol/mg) PGE-M/creatinine (pmol/mg)	209 210	1.35 74.2	0.93 142	0.06 9.82	0.16 0.74	6.60 1840	1.09 47.1	1.13 46.3

For PGE-M, in an earlier study, a total of 213 pooled smokers’ urine positive controls were embedded among 6379 urine samples (70 plates) for quality control purposes. The average PGE-M level for the positive controls was 36.8 ± 2.06 pmol/mL giving an inter-assay precision (% CV) of 5.6%. Intra-assay precision was determined by assaying 8 replicates of a pool of smokers’ urine. The mean and % CV were 35.1 pmol /mL and 3.5%, respectively. The accuracy of the PGE-M analysis was determined by analyzing a pooled non-smokers’ urine spiked in duplicate with 0, 38.1, 76.1, 152.3, 304.5 and 609 pmol PGE-M. These spiked samples were then processed using the method described here. The percent accuracies for the added levels were 110, 101, 103, 98.3 and 96.4. The average accuracy was 102%. The calculated LOD was ~ 1.1 pmol/mL.

**Fig 2:**
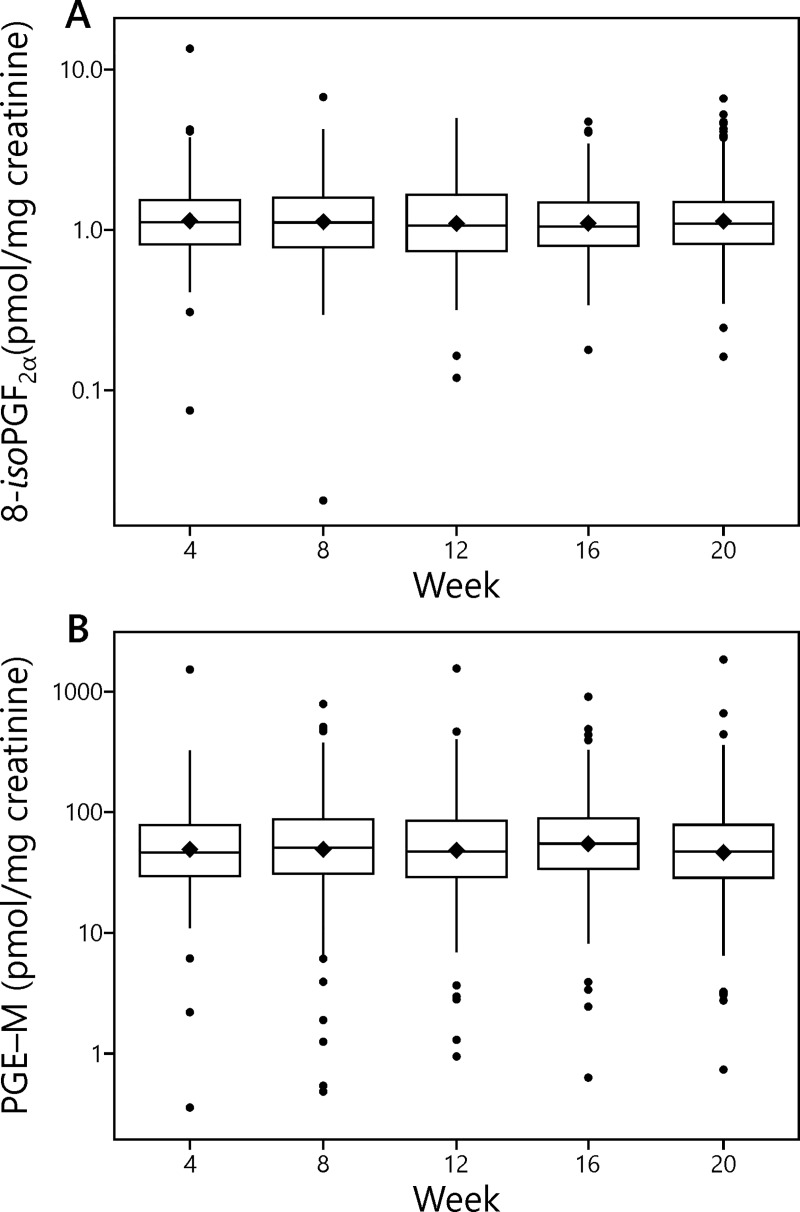
Mean creatinine-corrected values of 8-*iso*-PGF_2α_ and PGE-M at each week of the study in the subjects who smoked the Spectrum cigarettes. Horizontal line inside the box, median; black diamond, mean; bottom and top edge of the box, 1^st^ and 3^rd^ quartile (interquartile range [IQR]); the upper whisker extends from the top of the box to the largest value no further than 1.5 times IQR and the bottom whisker extends from the bottom of the box to the smallest value no further than 1.5 times IQR; the y-axis is in the natural log scale.

Means and other summary statistics for 8-*iso*-PGF_2α_ and PGE-M in this study are shown in **Table 1** and **Fig 2.** The mean level of 8-*iso*-PGF_2α_ at Week 4 was 1.34 ± 1.08 (S.D.) pmol/mg creatinine (N = 226) while that of PGE-M was 73.7 ± 113 (S.D.) pmol/mg creatinine (N = 232). The corresponding levels at Week 20 were 1.35 ± 0.93 (S.D.) pmol/mg creatinine (N = 209) for 8-*iso*-PGF_2α_ and 74.2 ± 142 (S.D.) pmol/mg creatinine (N = 210) for PGE-M. There was variation in these values in the intervening weeks.

Mean creatinine-corrected coefficients of variation for 8-*iso*-PGF_2α_ and PGE-M over the 20 week period were 31% and 54%, respectively, while the corresponding intra-class correlation coefficients (ICC) were 0.51 (95% CI, 0.45, 0.57) and 0.36 (0.30, 0.43), respectively. Exclusion of 21 subjects who reported having diabetes did not materially alter these results.

**Fig 3:**
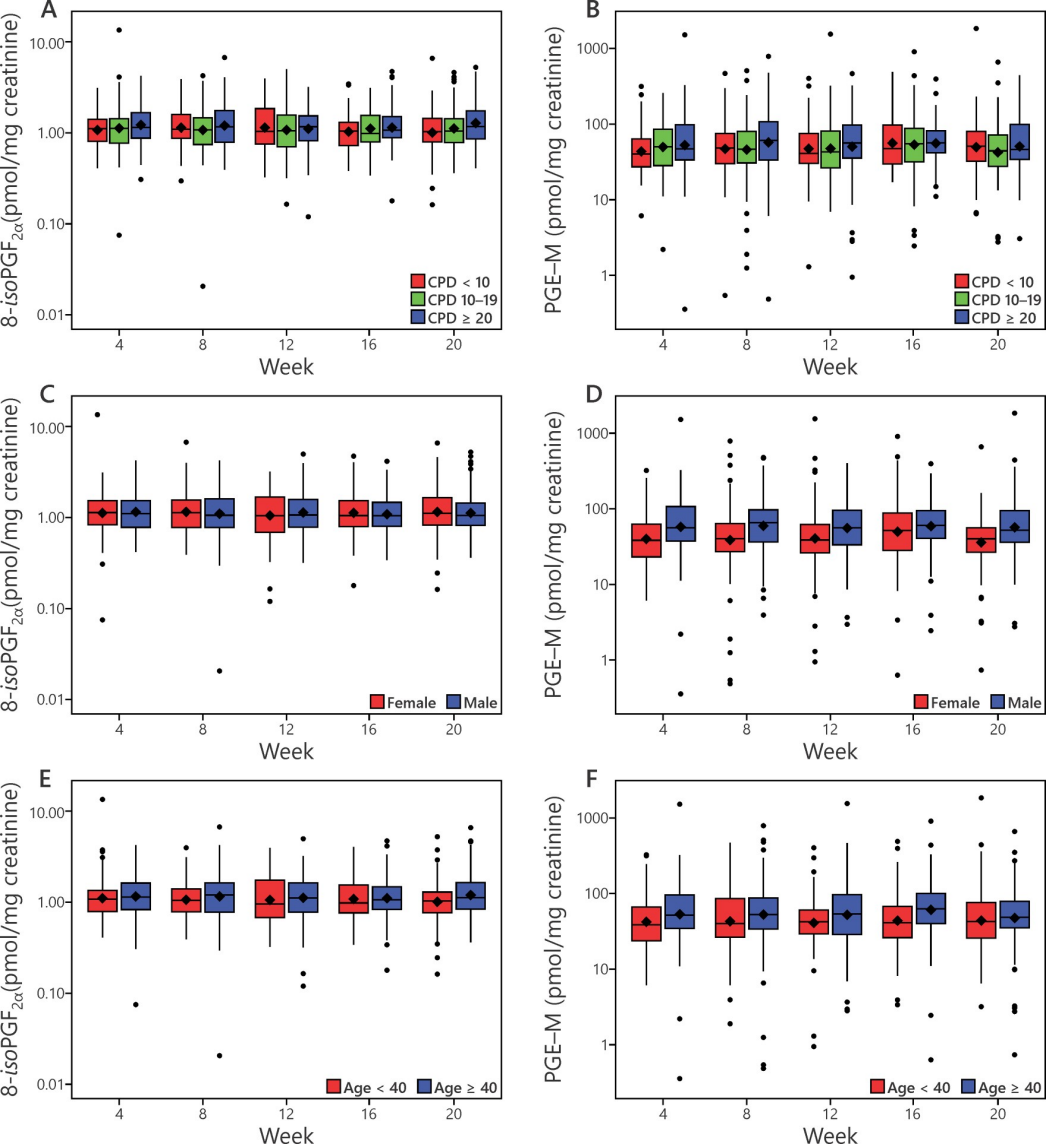
Effects of cigarettes per day (CPD) (panels A, B), gender (panels C, D), and age (panels E, F) on levels of 8-*iso*-PGF_2α_ and PGE-M. The effects of CPD on 8-*iso*-PGF_2α_ (p = 0.02), and gender (p<0.0001) and age (p<0.01) on PGE-M were significant. See also legend of **Fig 2**.

**Fig 4:**
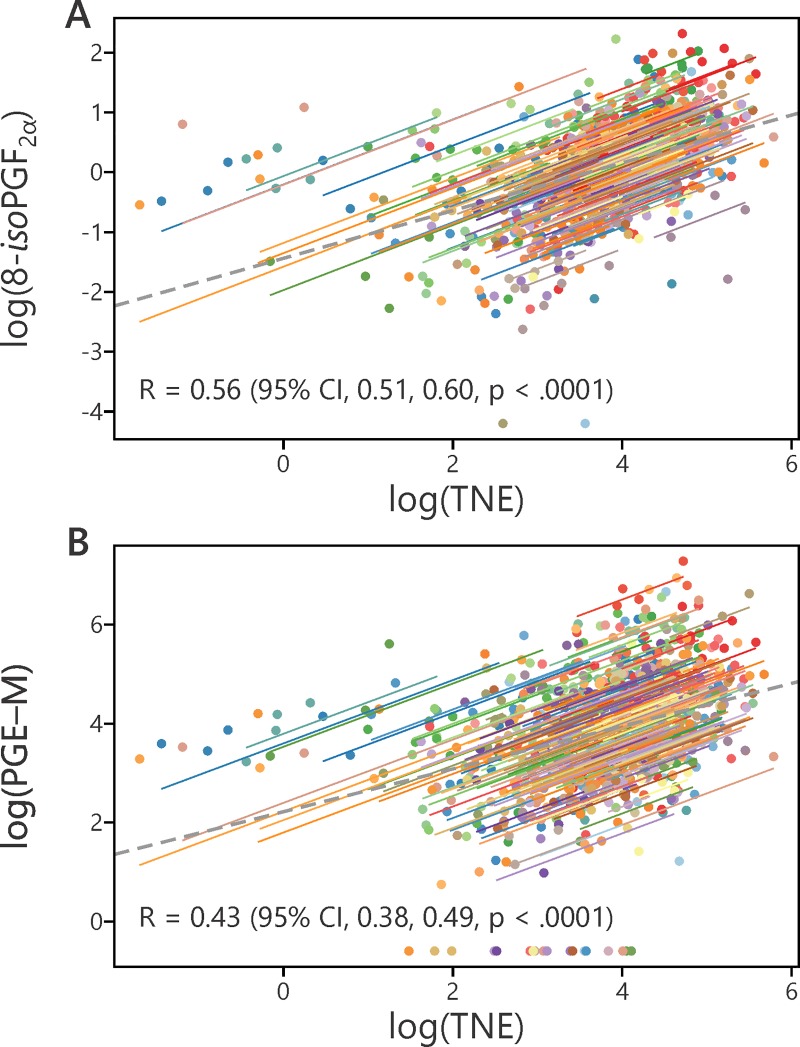
**A.** Correlation between log 8-*iso*-PGF_2α_ (pmol/mL) and log total nicotine equivalents (TNE) (nmol/mL) R = 0.56 (95% CI, 0.51, 0.60, p < .0001). **B.** Correlation between log PGE-M (pmol/mL) and log TNE (nmol/mL) R = 0.43 (95% CI, 0.38, 0.49, p < .0001).

Levels of 8-*iso*-PGF_2α_ and PGE-M were analyzed with respect to amount of smoking, gender, age, and BMI. Cigarettes smoked per day (CPD) had a significant enhancing effect (p = 0.02) on 8-*iso*-PGF_2α_ levels, but not on PGE-M (**Fig 3A and 3B**). Levels of 8-*iso*-PGF_2α_ and PGE-M also correlated significantly with TNE, p<0.0001 (**Fig 4A and 4B**). Males had significantly higher levels of PGE-M than females (p<0.0001), but there was no effect of gender on levels of 8-*iso*-PGF_2α_ (**Fig 3C and 3D**). Age had a significant effect on levels of PGE-M (p<0.01) but not on 8-*iso*-PGF_2α_ (**Fig 3E and 3F**). There were no significant effects of BMI on PGE-M and 8-*iso*-PGF_2α_.

The effects of CPD, gender, age, and BMI on ICC are summarized in **Table 2**. Males had higher ICC values than females for both 8-*iso*-PGF_2α_ (0.56, 95% CI [0.48, 0.64] for males vs. 0.46 [0.37, 0.56] for females) and PGE-M (0.43 [0.34, 0.51] for males vs 0.26 [0.18, 0.37] for females). This was confirmed by the lower mean CV observed in males than females for both 8-*iso*-PGF_2α_ (0.28 vs. 0.33, p = 0.03) and PGE-M (0.49 vs. 0.60, p<0.01). There were no significant CV differences related to age, BMI, or CPD for either biomarker.

**Table 2:** Influence of CPD, gender, age, and BMI on ICC values of 8-*iso*-PGF_2α_ and PGE-M.

Variable	Factor	Level	N of subjects	Creatinine corrected ICC (95% CI)
Log(8-*iso*PGF_2α_)	Gender	Female	103	0.46 (0.37, 0.56)
		Male	129	0.56 (0.48, 0.64)
	Age	<40	81	0.53 (0.42, 0.63)
		≥40	151	0.50 (0.42, 0.58)
	BMI	<25	79	0.44 (0.33, 0.55)
		25–29	77	0.57 (0.46, 0.67)
		≥30	76	0.54 (0.43, 0.64)
	CPD	<10	54	0.45 (0.32, 0.58)
		10–19	111	0.52 (0.43, 0.61)
		> = 20	67	0.54 (0.42, 0.65)
Log(PGE-M)	Gender	Female	103	0.26 (0.18, 0.37)
		Male	131	0.43 (0.34, 0.51)
	Age	<40	81	0.42 (0.31, 0.53)
		≥40	153	0.33 (0.26, 0.42)
	BMI	<25	80	0.40 (0.29, 0.51)
		25–29	78	0.36 (0.26, 0.48)
		≥30	76	0.32 (0.22, 0.45)
	CPD	<10	54	0.29 (0.17, 0.44)
		10–19	112	0.39 (0.30, 0.49)
		> = 20	68	0.37 (0.26, 0.50)

## Discussion

The analytical chemistry validation data reported here were important for our study of the longitudinal stability of 8-*iso*-PGF_2α_ and PGE-M. Although these LC-MS/MS methods are well described and validated in the literature [7, 23], there are some differences between the methods we used and the originally described versions. For 8-*iso*-PGF_2α_, we used one tenth the amount of urine and a different solid-phase extraction and high throughput 96-well plate protocol. For both 8-*iso*-PGF_2α_ and PGE-M, the LC-MS/MS conditions were also somewhat different from those described previously. The validation parameters reported here support the accuracy and precision of our methods.

Our results do not support good longitudinal within subject stability of 8-*iso*-PGF_2α_ and PGE-M levels in cigarette smokers. The ICC values of 0.51 for 8-*iso*-PGF_2α_ and 0.36 for PGE-M are relatively low. Cicchetti *et al* have described ICC values between 0.40 and 0.59 as “fair” which would be the appropriate descriptor for 8-*iso*-PGF_2α_ in this study while the PGE-M value is even lower than that category [28]. Our results are quite different from those of Wu *et al* who reported ICC values of 0.76 and 0.67 for 8-*iso*-PGF_2α_ and PGE-M, respectively [21]. Unlike our study, they measured the biomarkers in 48 randomly chosen participants in the Shanghai Men’s Health Study. We note that our ICC values are higher in males than in females; 0.56 for 8-*iso*-PGF_2α_ and 0.43 for PGE-M, which however are still lower than those reported by Wu *et al* for males only. Their samples were collected in each season over the period of one year, whereas ours were collected over a 20 week period [21]. Their study was comprised of 56% smokers whereas all of our participants were smokers. All of our participants smoked Spectrum cigarettes which have characteristics similar to that of a typical American blend whereas their study participants presumably smoked predominantly Chinese cigarettes [29]. The intra-individual consistency of the biomarkers observed in the Shanghai study may also have been inflated by greater stability of the biomarkers in non-smokers. Our data indicate that smoking contributes to the longitudinal instability of PGE-M because correction for both creatinine and total nicotine equivalents results in an improved ICC value of 0.47 (0.40, 0.53).

The levels of 8-*iso*-PGF_2α_ and PGE-M observed here would reflect the balance between the formation of these endogenous compounds due to oxidative damage and inflammation and their metabolism and pharmacokinetics. Pharmacokinetic studies indicate that 8-*iso*-PGF_2α_ is rapidly metabolized, as demonstrated by Roberts *et al* [30] who showed that 75% of radioactive 8-*iso*-PGF_2α_ infused into a human was excreted in the urine in 4.5h, consistent with the rapid metabolism of this compound observed in rabbits (plasma half-life, 1 min) [31]. A steady state between formation due to the effects of cigarette smoking among other factors and metabolism and excretion of 8-*iso*-PGF_2α_ and PGE-M would presumably be reached in the smokers in this study, but other factors such as diet and toxicant exposure can also result in both oxidative damage and inflammation. While 8-*iso*-PGF_2α_ is widely considered as the premier biomarker for detection of chemical lipid peroxidation, it has been noted that biosynthesis of 8-*iso*-PGF_2α_ via enzymatic lipid peroxidation by prostaglandin-endoperoxide synthases, which are induced in inflammation, could also contribute, thus influencing its longitudinal stability [32].

The influence of smoking on 8-*iso*-PGF_2α_ and PGE-M levels has been assessed in smoking cessation studies. In one recent study, we found that when cigarette smokers stopped smoking for 12 weeks, their levels of 8-*iso*-PGF_2α_ decreased significantly but modestly (27% reduction) from 1.05 pmol/mg creatinine to 0.73 pmol/mg creatinine [33]. This is similar to the value of 0.83 pmol/mg creatinine of 8-*iso*-PGF_2α_ reported in a study of non-smokers using essentially the same LC-MS/MS methodology [7]. For PGE-M, a significant but modest decrease of 44% was observed when cigarette smokers stopped smoking, to 43.1 pmol/mg creatinine, similar to a value of 30.2 pmol/mg creatinine which has been reported for non-smokers [17, 33]. These decreases in biomarker levels are far less than observed for typical biomarkers of exposure, which decrease by 39–92% within only 3 days after smoking cessation [34]. While biomarkers of exposure depend mainly on specific toxicant exposures which stop upon smoking cessation, biomarkers such as 8-*iso*-PGF_2α_ and PGE-M, which have been termed “biomarkers of potential harm” [4], result from alterations in physiologic processes related to oxidative damage and inflammation [35, 36]. PGE-M has also been described as a potential cancer biomarker and its levels have been positively associated with high saturated fat intake, obesity, and poor self-reported health status in addition to cigarette smoking and other factors [37]. Thus, the relatively modest longitudinal stabilities in cigarette smokers of 8-*iso*-PGF_2α_ and PGE-M are undoubtedly explained by the multiple endogenous and exogenous factors which contribute to their levels.

We observed significant correlations of both log 8-*iso*-PGF_2α_ and log PGE-M with log TNE. Since TNE is a gold standard specific biomarker of cigarette smoke exposure in this study, these results are fully consistent with the conclusion that cigarette smoking induces both oxidative damage and inflammation. The correlation coefficients could be affected by factors other than cigarette smoking that induce oxidative damage and inflammation, as noted above, or by the different pharmacokinetic profiles of TNE, 8-*iso*-PGF_2α_ and PGE-M.

We observed that males had higher ICC values than females for both 8-*iso*-PGF_2α_ and PGE-M. While the explanation for this observation is not clear, it is unlikely to be due to time since last cigarette because all urine samples in this study were first morning voids.

A strength of this study is that the participants smoked mainly one type of cigarette, provided to them free of charge, over the 20 week course of the study. This represents a unique opportunity to test the longitudinal stability of biomarkers in cigarette smokers. Separately, we have assessed the longitudinal stability of biomarkers of acrylonitrile, acrolein, and nicotine exposure, and find ICCs of 0.67, 0.46, and 0.68, respectively, considerably higher than reported here [38]. This undoubtedly results from the fact that these are biomarkers mainly of cigarette smoke exposure as opposed to the biomarkers of potential harm quantified here, for which multiple non-cigarette sources may contribute. There were several potential limitations of the study. The study duration of 20 weeks was somewhat limited and the study cigarettes do not represent fully all cigarettes that are available in the marketplace. Smoking of the Spectrum cigarettes was based only on subjects’ self-report. Also, the applicability of the study results to the general population of smokers is uncertain.

In summary, using a unique study design, we have demonstrated fair longitudinal stability in cigarette smokers for the widely used oxidative damage biomarker 8-*iso*-PGF_2α_. In contrast, we observed rather poor longitudinal stability for the inflammation biomarker PGE-M. The variation of these biomarkers in cigarette smokers over the 20 week study period undoubtedly is related to the multiple endogenous and exogenous factors that can influence oxidative damage and inflammation.

## Supporting information

S1 Supporting Information Statistical analysis of longitudinal stability of 8-*iso*-PGF_2α_ and PGE-M (data analyzed, statistical methods). (PDF)

S2 Supporting Information Summary of variables by week. (PDF)

S3 Supporting Information Coefficients of variation and ICC corrected by various parameters. (PDF)

S4 Supporting Information Correlation between log 8-*iso*-PGF_2a_ and log total nicotine equivalents (TNE). (PDF)

S5 Supporting Information Correlation between log PGE-M and log TNE. (PDF)

S6 Supporting Information Repeated measures analysis. (PDF)

S7 Supporting Information ICC for subgroups defined by different factors. (PDF)

S8 Supporting Information Boxplots of biomarker levels by week and each covariate. (PDF)

S9 Supporting Information Coefficients of variation by gender, age, BMI, and cigarettes per day. (PDF)

S10 Supporting Information Scatter plots of CV for continuous age, BMI, and CPD. (PDF)

S11 Supporting Information Bivariate and multivariable linear regression of CV of each biomarker on gender, age, BMI, and cigarettes per day. (PDF)

S12 Supporting Information Related analysis for C-Reactive protein (CRP). (PDF)
