# Supplementary material for: Longitudinal stability in cigarette smokers of urinary eicosanoid biomarkers of oxidative damage and inflammation
Source: PLoS One. 2019 Apr 25;14(4):e0215853. doi: 10.1371/journal.pone.0215853 (PMC6483352; doi:10.1371/journal.pone.0215853)
Supplement: S1 Supporting Information — (PDF) [file pone.0215853.s001.pdf]

S1 Supporting Information. Statistical analysis of longitudinal stability of 8-*iso*-$PGF_{2\alpha}$ and PGE-M (Data Analyzed, Statistical Methods).

Statistical Analysis of Longitudinal Stability of 8-*iso*-$PGF_{2\alpha}$ and PGE-M
Statistician: Xianghua Luo, PhD
Version:

- (8/10/2018) Longitudinal stability for the overall group
- (8/30/2018) Gender difference in these biomarkers at each time point and difference in longitudinal stability during weeks 4-20 between two genders
- (9/16/2018) Difference in these biomarkers (and longitudinal stability) for different BMI, age, amount of smoking groups.
- (10/22/2018) Added coefficient of variation.
- (11/11/2018) Some additional analyses were added for the 1st draft of the manuscript
- (11/22/2018) CRP analysis is added.

8/10/2018
Data analyzed

1. Only control subjects' data collected at visits 4, 8, 12, 16, and 20 were used. Note that I excluded visit 0 because at visit 0 control subjects used their own brand cigarettes, while at visits 4-20 they used study cigarettes. Let me know if you want me to add back visit 0.
2. I analyzed creatinine-corrected, uncorrected, TNE-corrected, and creatinine-and-TNE-corrected values. The corrected values and their unit are defined as follows:
   - Non-corrected: pmol/mL urine.
     Note:
     - Based on Steve Carmella's email on 9/8/2017, the unit in the raw data should be pmol/mL of urine, not nmol/mL as indicated in the Excel datasheet.
     - 8-isoPGF2a (pmol/mL), non-detectable imputed with 1/2 LOD (=0.5*0.03=0.015)
     - PGEM (pmol/mL), non-detectable imputed with 1/2 LOD (=0.5*1.1=0.55)
   - Creatinine-corrected (pmol/mg creatinine): non-corrected value (pmol/mL) divided by creatinine (mg/dL), and then times 100;
   - TNE-corrected ($\times 10^3$): non-corrected value (pmol/mL) divided by TNE (nmol/mL);
   - Creatinine-and-TNE-corrected (μL/mg of creatinine): creatinine-corrected value (pmol/mg of creatinine) divided by TNE (nmol/mL).

Statistical Methods
Following Hankinson et. al. (1995), intraclass correlation coefficient (ICC) and 95% confidence intervals (CI) were estimated using the SAS macro %ICC9 (Hertzmark and Spiegelman, 2010), where linear mixed model for log-transformed biomarker was used with a random intercept. Note that missing was assumed to be at random.
Coefficient of variation (CV) for each control participant who had ≥ 2 measurements was calculated as the sample standard deviation divided by the sample mean. Summary statistics of CV, including N, mean, median, interquartile range (25% and 75% percentiles), and range (minimum and maximum), are presented. Note that higher ICC and lower CV correspond to better longitudinal stability.
The correlations of log-transformed biomarkers, 8-isoPGF2a (pmol/mL) and TNE (nmol/mL) and PGEM (pmol/mL) and TNE (nmol/mL), in the control subjects were estimated by using the R package rmcorr (Bakdash and Marusich, 2018). Note that the biomarkers in this correlation analysis were not corrected by creatinine. The scatterplot of two biomarkers was presented, with a different color used for each subject and parallel lines are fitted to each subject's data. Note that it doesn't matter whether the two variables for calculating correction have the same unit or not because in log scale, variables with different units are only different by a constant, which does not affect correlation.
