# Supplementary material for: Longitudinal stability in cigarette smokers of urinary eicosanoid biomarkers of oxidative damage and inflammation
Source: PLoS One. 2019 Apr 25;14(4):e0215853. doi: 10.1371/journal.pone.0215853 (PMC6483352; doi:10.1371/journal.pone.0215853)
Supplement: S2 Supporting Information — (PDF) [file pone.0215853.s002.pdf]

S2 Supporting Information. Summary of variables by week.

Summary of variables by week
Creatinine corrected

Week	Variable	N	Mean	Std Dev	Std Error	Min	Max	Median	Geometric Mean
4	8-isoPGF$_{2\alpha}$/creatinine (pmol/mg)	226	1.340	1.077	0.072	0.075	13.469	1.115	1.140
	PGEM/creatinine (pmol/mg)	232	73.702	112.482	7.385	0.356	1519.679	46.179	49.032
8	8-isoPGF$_{2\alpha}$ /creatinine (pmol/mg)	215	1.317	0.831	0.057	0.021	6.717	1.112	1.124
	PGEM/creatinine (pmol/mg)	222	74.777	88.524	5.941	0.485	788.402	50.873	49.188
12	8-isoPGF$_{2\alpha}$ /creatinine (pmol/mg)	210	1.279	0.736	0.051	0.120	4.976	1.063	1.097
	PGEM/creatinine (pmol/mg)	217	75.432	121.070	8.219	0.945	1551.640	47.215	48.260
16	8-isoPGF$_{2\alpha}$ /creatinine (pmol/mg)	206	1.260	0.731	0.051	0.179	4.721	1.048	1.101
	PGEM/creatinine (pmol/mg)	212	80.698	92.232	6.335	0.631	905.530	54.871	54.752
20	8-isoPGF$_{2\alpha}$ /creatinine (pmol/mg)	209	1.348	0.928	0.064	0.162	6.587	1.092	1.134
	PGEM/creatinine (pmol/mg)	210	74.232	142.324	9.821	0.736	1841.490	47.111	46.323

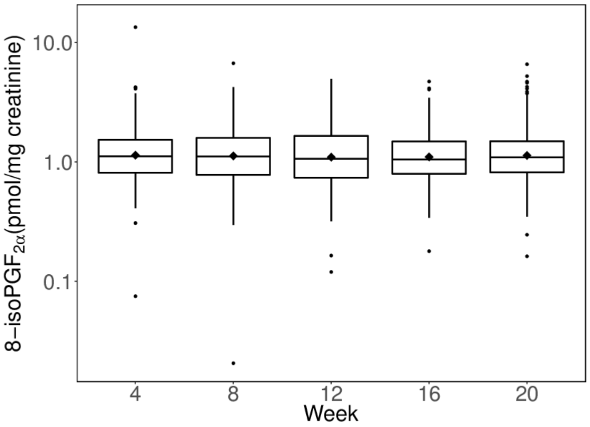

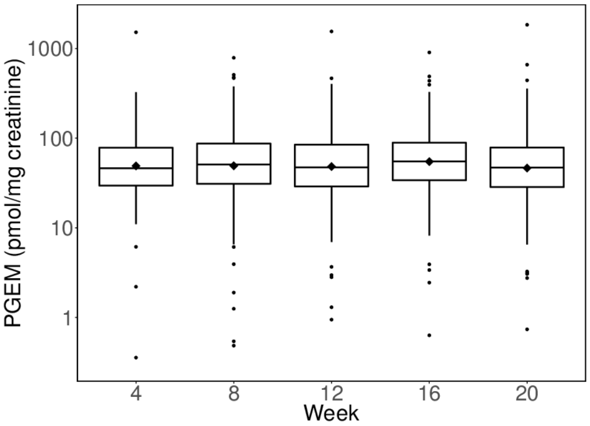

Horizontal line inside the box: median; black diamond: mean; bottom and top edge of the box: 1st and 3rd quartile (interquartile range [IQR]); the upper whisker extends from the top of the box to the largest value no further than 1.5 times IQR and the bottom whisker extends from the bottom of the box to the smallest value no further than 1.5 times IQR; the y-axis is in natural log scale.

Non-corrected

Week	Variable	N	Mean	Std Dev	Std Error	Minimum	Maximum
4	8-isoPGF$_{2\alpha}$ (pmol/mL)	226	1.470	1.073	0.071	0.015	7.481
	PGEM (pmol/mL)	232	83.745	108.804	7.143	0.550	823.833
8	8-isoPGF$_{2\alpha}$ (pmol/mL)	215	1.398	1.070	0.073	0.015	7.916
	PGEM (pmol/mL)	222	84.109	133.705	8.974	0.550	1458.530
12	8-isoPGF$_{2\alpha}$ (pmol/mL)	210	1.421	1.153	0.080	0.094	7.576
	PGEM (pmol/mL)	217	82.577	107.059	7.268	0.550	1040.256
16	8-isoPGF$_{2\alpha}$ (pmol/mL)	206	1.405	1.220	0.085	0.072	9.259
	PGEM (pmol/mL)	212	89.713	107.094	7.355	0.550	756.763
20	8-isoPGF$_{2\alpha}$ (pmol/mL)	209	1.468	1.225	0.085	0.101	10.134
	PGEM (pmol/mL)	210	84.515	118.297	8.163	0.550	834.797

TNE corrected

Week	Variable	N	Mean	Std Dev	Std Error	Minimum	Maximum
4	8-isoPGF$_{2\alpha}$ (TNE corrected, $\times 10^3$)	226	0.053	0.208	0.014	0.000	2.317
	PGEM (TNE corrected, $\times 10^3$)	232	2.310	6.555	0.430	0.012	78.407
8	8-isoPGF$_{2\alpha}$ (TNE corrected, $\times 10^3$)	215	0.046	0.189	0.013	0.001	2.278
	PGEM (TNE corrected, $\times 10^3$)	222	2.410	8.055	0.541	0.009	92.530
12	8-isoPGF$_{2\alpha}$ (TNE corrected, $\times 10^3$)	210	0.078	0.523	0.036	0.001	7.345
	PGEM (TNE corrected, $\times 10^3$)	217	2.925	10.390	0.705	0.010	112.053
16	8-isoPGF$_{2\alpha}$ (TNE corrected, $\times 10^3$)	206	0.046	0.151	0.011	0.003	1.798
	PGEM (TNE corrected, $\times 10^3$)	212	2.659	6.766	0.465	0.016	76.976
20	8-isoPGF$_{2\alpha}$ (TNE corrected, $\times 10^3$)	209	0.067	0.288	0.020	0.002	3.166
	PGEM (TNE corrected, $\times 10^3$)	210	3.709	15.315	1.057	0.025	153.810

Creatinine-and-TNE-corrected

Week	Variable	N	Mean	Std Dev	Std Error	Minimum	Maximum
4	8-isoPGF$_{2\alpha}$ (uL/mg, creatinine and TNE corrected)	226	0.060	0.200	0.013	0.002	2.370
	PGEM (uL/mg, creatinine and TNE corrected)	232	4.108	28.917	1.899	0.008	435.596
8	8-isoPGF$_{2\alpha}$ (uL/mg, creatinine and TNE corrected)	215	0.051	0.142	0.010	0.001	1.416
	PGEM (uL/mg, creatinine and TNE corrected)	222	3.239	13.544	0.909	0.008	181.541
12	8-isoPGF$_{2\alpha}$ (uL/mg, creatinine and TNE corrected)	210	0.088	0.569	0.039	0.001	8.036
	PGEM (uL/mg, creatinine and TNE corrected)	217	3.729	13.076	0.888	0.017	122.605
16	8-isoPGF$_{2\alpha}$ (uL/mg, creatinine and TNE corrected)	206	0.058	0.170	0.012	0.002	1.961
	PGEM (uL/mg, creatinine and TNE corrected)	212	4.062	14.545	0.999	0.018	158.039
20	8-isoPGF$_{2\alpha}$ (uL/mg, creatinine and TNE corrected)	209	0.071	0.280	0.019	0.002	3.457
	PGEM (uL/mg, creatinine and TNE corrected)	210	4.394	19.955	1.377	0.090	204.385
