# Supplementary material for: Longitudinal stability in cigarette smokers of urinary eicosanoid biomarkers of oxidative damage and inflammation
Source: PLoS One. 2019 Apr 25;14(4):e0215853. doi: 10.1371/journal.pone.0215853 (PMC6483352; doi:10.1371/journal.pone.0215853)
Supplement: S3 Supporting Information — (PDF) [file pone.0215853.s003.pdf]

S3 Supporting Information. Coefficients of variation and ICC corrected by various parameters.

Coefficients of variation (CV)

Creatinine-corrected

Variable	N	Mean	Median	25th Pctl	75th Pctl	Minimum	Maximum
8-isoPGF$_{2\alpha}$ /creatinine (pmol/mg)	222	0.31	0.17	0.28	0.19	0.39	0.03
PGEM/creatinine (pmol/mg)	224	0.54	0.32	0.47	0.33	0.68	0.11

Non-corrected

Variable	N	Mean	Median	25%	75%	Min	Max
8-isoPGF$_{2\alpha}$ (pmol/mL)	222	0.45	0.21	0.43	0.29	0.59	0.06
PGEM (pmol/mL)	224	0.65	0.29	0.59	0.45	0.80	0.07

TNE-corrected

Variable	N	Mean	Median	25%	75%	Min	Max
8-isoPGF$_{2\alpha}$ (TNE corrected, ×10^3)	222	0.42	0.30	0.33	0.24	0.51	0.09
PGEM (TNE corrected, ×10^3)	224	0.61	0.37	0.50	0.37	0.74	0.01

Creatinine-and-TNE-corrected

Variable	N	Mean	Median	25%	75%	Min	Max
8-isoPGF$_{2\alpha}$ (uL/mg, creatinine and TNE corrected)	222	0.63	0.34	0.56	0.4	0.76	0.04
PGEM (uL/mg, creatinine and TNE corrected)	224	0.73	0.41	0.65	0.44	0.89	0.04

Conclusion

8-epiPGF$_{2\alpha}$ has smaller CV (better stability) than PGEM, regardless of the correction.

Estimated ICC based on the mixed model of log-transformed values

Variable	N	Creatinine corrected ICC (95% CI)	Non corrected ICC (95% CI)	TNE corrected ICC (95% CI)	Creatinine and TNE corrected ICC (95% CI)
8-isoPGF$_{2\alpha}$	232	0.51 (0.45, 0.57)	0.42 (0.36, 0.49)	0.59 (0.53, 0.65)	0.51 (0.45, 0.57)
PGEM	234	0.36 (0.30, 0.43)	0.41 (0.35, 0.48)	0.53 (0.47, 0.59)	0.47 (0.40, 0.53)

Conclusion

8-isoPGF$_{2\alpha}$ has higher ICC (better stability) than PGEM, regardless of the correction.
