# Supplementary material for: Longitudinal stability in cigarette smokers of urinary eicosanoid biomarkers of oxidative damage and inflammation
Source: PLoS One. 2019 Apr 25;14(4):e0215853. doi: 10.1371/journal.pone.0215853 (PMC6483352; doi:10.1371/journal.pone.0215853)

S4 Supporting Information. Correlation between log 8-*iso*-PGF_{2a} and log total nicotine equivalents (TNE).

Correlation between log 8-iso$PGF_{2\alpha}$ (pmol/mL) and log TNE (nmol/mL)
R=0.56 (95% CI, 0.51, 0.60, p<.0001).

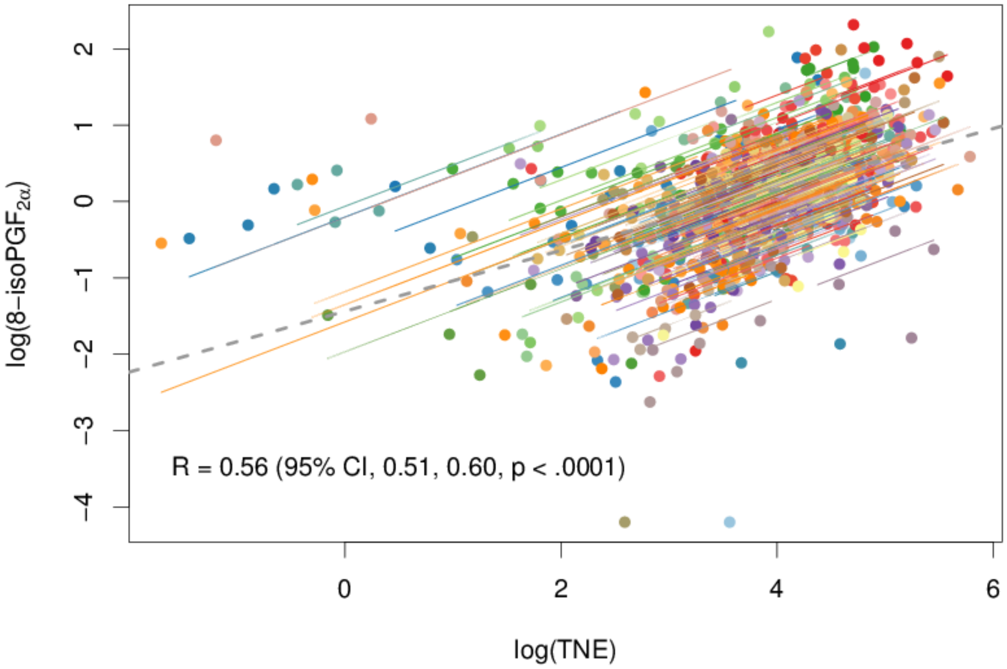

Supplement: S4 Supporting Information — (PDF) [file pone.0215853.s004.pdf]
