# Supplementary material for: Longitudinal stability in cigarette smokers of urinary eicosanoid biomarkers of oxidative damage and inflammation
Source: PLoS One. 2019 Apr 25;14(4):e0215853. doi: 10.1371/journal.pone.0215853 (PMC6483352; doi:10.1371/journal.pone.0215853)
Supplement: S5 Supporting Information — (PDF) [file pone.0215853.s005.pdf]

S5 Supporting Information. Correlation between log PGE-M and log TNE.

Correlation between log PGEM (pmol/mL) and log TNE (nmol/mL)
R=0.43 (95% CI, 0.38, 0.49, p<.0001).

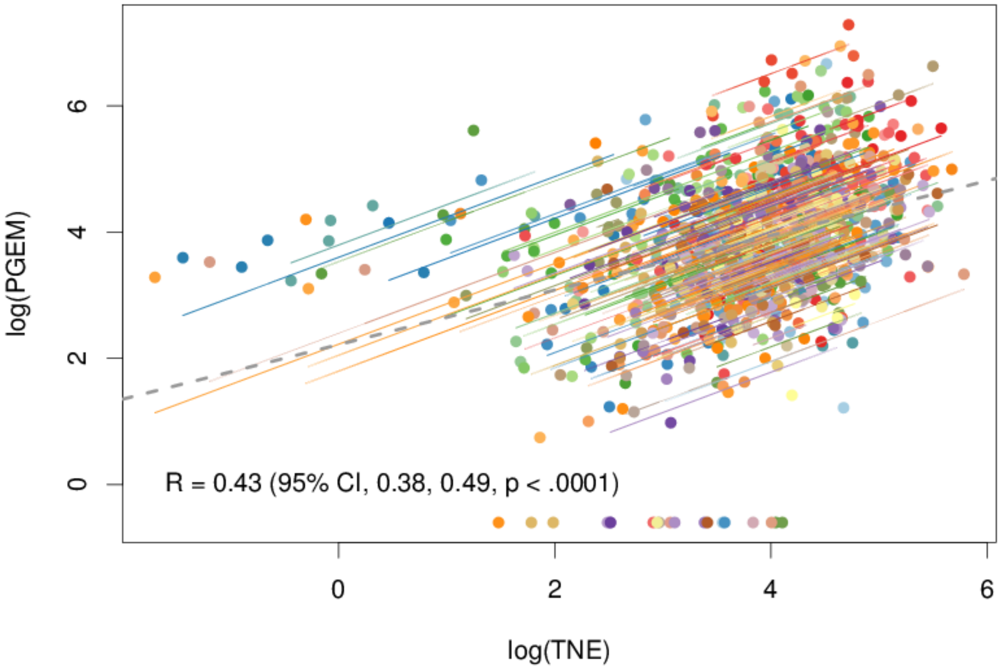

Reference
Hankinson SE, Manson JE, Spiegelman D, Willett WC, Longcope C, and Speizer FE. (1995). Reproducibility of plasma hormone levels in postmenopausal women over a 2-3-year period. Cancer Epidemiology, Biomarkers & Prevention, 4: 649-654.
Hertzmark E and Spiegelman D. (2010). The SAS ICC9 macro. https://www.hsph.harvard.edu/donna-spiegelman/software/icc9/
(If the 95% CI of CV is needed, then need the following reference)
Bland JM, Altman DG. (1996). Measurement error proportional to the mean. BMJ, 313:106-106.
Bland JM. How should I calculate a within-subject coefficient of variation? https://www-users.york.ac.uk/~mb55/meas/cv.htm (accessed on January 23, 2018).
Bakdash JZ and Marusich LR. (2017). Repeated measures correlation. Front Psychol. 2017; 8: 456.
