# Supplementary material for: Longitudinal stability in cigarette smokers of urinary eicosanoid biomarkers of oxidative damage and inflammation
Source: PLoS One. 2019 Apr 25;14(4):e0215853. doi: 10.1371/journal.pone.0215853 (PMC6483352; doi:10.1371/journal.pone.0215853)
Supplement: S6 Supporting Information — (PDF) [file pone.0215853.s006.pdf]

S6 Supporting Information. Repeated measures analysis.

9/16/2018

Repeated measures analysis
Gender, body mass index (BMI), age, and amount of smoking at randomization were analyzed in terms of their association with the level of the biomarkers and the longitudinal stability of these biomarkers. The repeated measures of log-transformed biomarkers during weeks 4-20 were analyzed for each factor (e.g., gender) using a linear mixed model with a fixed effect of the studied factor and a random intercept. The p-value of the fixed effect of the studied factor is reported.

**Variable**	**Factor**	**Creatinine corrected**	**Non corrected**	**TNE corrected**	**Creatinine and TNE corrected**
		Estimated fixed effect (95% CI), p-value	Estimated fixed effect (95% CI), p-value	Estimated fixed effect (95% CI), p-value	Estimated fixed effect (95% CI), p-value
Log(8-isoPGF$_{2\alpha}$)	Female	-0.01 (-0.13, 0.10) p = 0.81	-0.27 (-0.42, -0.12) **p < 0.001**	-0.06 (-0.25, 0.14) p = 0.56	0.20 (-0.02, 0.42) p = 0.07
	Age (×10 years)	0.03 (-0.02, 0.07) p = 0.24	-0.10 (-0.16, -0.05) **p < 0.001**	-0.12 (-0.19, -0.05) **p < 0.001**	0.01 (-0.08, 0.09) p = 0.89
	BMI (kg/m^2)	0.00 (-0.01, 0.01) p = 0.51	0.01 (-0.01, 0.02) p = 0.30	0.01 (-0.00, 0.03) p = 0.051	0.01 (-0.01, 0.03) p = 0.20
	CPD (×10)	0.16 (0.03, 0.30) **p = 0.02**	0.02 (-0.17, 0.20) p = 0.87	-0.59 (-0.81, -0.37) **p < 0.0001**	-0.44 (-0.70, 0.19) **p < 0.001**
Log(PGEM)	Female	-0.35 (-0.52, -0.18) **p < 0.0001**	-0.62 (-0.82, -0.41) **p < 0.0001**	-0.39 (-0.63, -0.14) **p < 0.01**	-0.12 (-0.38, 0.13) p = 0.35
	Age (×10 years)	0.09 (0.02, 0.15) **p < 0.01**	-0.04 (-0.13, 0.04) p = 0.31	-0.06 (-0.15, 0.03) p = 0.20	0.07 (-0.03, 0.16) p = 0.16
	BMI (kg/m^2)	-0.01 (-0.02, 0.01) p = 0.31	-0.00 (-0.02, 0.01) p = 0.71	0.00 (-0.01, 0.02) p = 0.62	0.00 (-0.02, 0.02) p = 0.92
	CPD (×10)	0.04 (-0.16, 0.25) p = 0.69	-0.09 (-0.36, 0.17) p = 0.50	-0.71 (-1.00, -0.43) **p < 0.0001**	-0.58 (-0.88, -0.28) **p < 0.001**
