# Supplementary material for: Longitudinal stability in cigarette smokers of urinary eicosanoid biomarkers of oxidative damage and inflammation
Source: PLoS One. 2019 Apr 25;14(4):e0215853. doi: 10.1371/journal.pone.0215853 (PMC6483352; doi:10.1371/journal.pone.0215853)
Supplement: S7 Supporting Information — (PDF) [file pone.0215853.s007.pdf]

S7 Supporting Information. ICC for subgroups defined by different factors.

ICC for subgroups defined by different factors

Variable	Factor	Level	N of subjects	Creatinine corrected ICC (95% CI)	Non corrected ICC (95% CI)	TNE corrected ICC (95% CI)	Creatinine and TNE corrected ICC (95% CI)
Log(8-isoPGF$_{2\alpha}$)	Gender	Female	103	0.46 (0.37, 0.56)	0.39 (0.29, 0.49)	0.65 (0.56, 0.72)	0.50 (0.41, 0.59)
		Male	129	0.56 (0.48, 0.64)	0.42 (0.34, 0.51)	0.49 (0.40, 0.57)	0.51 (0.42, 0.59)
	Age	<40	81	0.53 (0.42, 0.63)	0.27 (0.17, 0.39)	0.55 (0.45, 0.65)	0.48 (0.37, 0.59)
		≥40	151	0.50 (0.42, 0.58)	0.46 (0.38, 0.54)	0.61 (0.54, 0.68)	0.52 (0.45, 0.60)
	BMI	<25	79	0.44 (0.33, 0.55)	0.37 (0.26, 0.49)	0.56 (0.46, 0.66)	0.50 (0.40, 0.61)
		25-29	77	0.57 (0.46, 0.67)	0.48 (0.37, 0.59)	0.60 (0.50, 0.69)	0.48 (0.37, 0.59)
		≥30	76	0.54 (0.43, 0.64)	0.44 (0.33, 0.55)	0.61 (0.51, 0.70)	0.52 (0.42, 0.63)
	CPD	<10	54	0.45 (0.32, 0.58)	0.30 (0.18, 0.45)	0.54 (0.41, 0.66)	0.46 (0.33, 0.59)
		10-19	111	0.52 (0.43, 0.61)	0.42 (0.33, 0.51)	0.50 (0.41, 0.59)	0.46 (0.37, 0.56)
		>=20	67	0.54 (0.42, 0.65)	0.52 (0.40, 0.64)	0.64 (0.54, 0.73)	0.52 (0.40, 0.63)
Log(PGEM)	Gender	Female	103	0.26 (0.18, 0.37)	0.24 (0.16, 0.34)	0.47 (0.38, 0.57)	0.41 (0.32, 0.51)
		Male	131	0.43 (0.34, 0.51)	0.46 (0.37, 0.55)	0.57 (0.49, 0.64)	0.53 (0.44, 0.61)
	Age	<40	81	0.42 (0.31, 0.53)	0.35 (0.25, 0.48)	0.49 (0.38, 0.60)	0.44 (0.33, 0.55)
		≥40	153	0.33 (0.26, 0.42)	0.43 (0.35, 0.51)	0.55 (0.47, 0.62)	0.47 (0.40, 0.55)
	BMI	<25	80	0.40 (0.29, 0.51)	0.45 (0.34, 0.56)	0.55 (0.44, 0.65)	0.46 (0.35, 0.57)
		25-29	78	0.36 (0.26, 0.48)	0.42 (0.32, 0.54)	0.51 (0.41, 0.62)	0.45 (0.34, 0.56)
		≥30	76	0.32 (0.22, 0.45)	0.34 (0.24, 0.47)	0.53 (0.42, 0.63)	0.49 (0.38, 0.60)
	CPD	<10	54	0.29 (0.17, 0.44)	0.35 (0.23, 0.50)	0.50 (0.37, 0.63)	0.40 (0.28, 0.54)
		10-19	112	0.39 (0.30, 0.49)	0.41 (0.32, 0.51)	0.47 (0.38, 0.56)	0.42 (0.33, 0.52)
		>=20	68	0.37 (0.26, 0.50)	0.43 (0.32, 0.55)	0.54 (0.43, 0.65)	0.50 (0.38, 0.61)

Conclusions:

- Males had higher longitudinal stability than females in all biomarkers, except for TNE-corrected 8-isoPGF$_{2\alpha}$.
- Heavy smokers had higher stability in all biomarkers than light smokers.
- Age and BMI's relationship with ICC is not obvious.

Note: there is no formal test of p-value associated with these ICC comparisons. Please see the CV regression analysis and p-value toward the end of this report.
