# Supplementary material for: Longitudinal stability in cigarette smokers of urinary eicosanoid biomarkers of oxidative damage and inflammation
Source: PLoS One. 2019 Apr 25;14(4):e0215853. doi: 10.1371/journal.pone.0215853 (PMC6483352; doi:10.1371/journal.pone.0215853)
Supplement: S8 Supporting Information — (PDF) [file pone.0215853.s008.pdf]

S8 Supporting Information. Boxplots of biomarker levels by week and each covariate.

Boxplots of biomarker levels by week and each covariate

<u>Creatinine corrected</u>

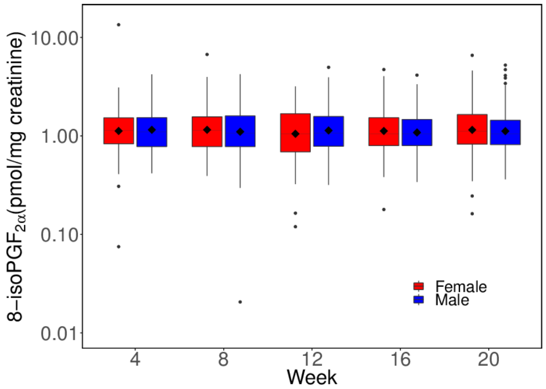

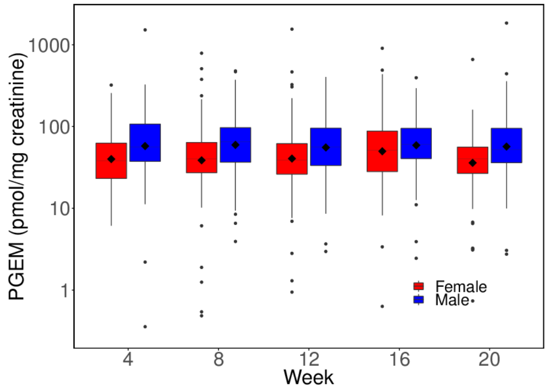

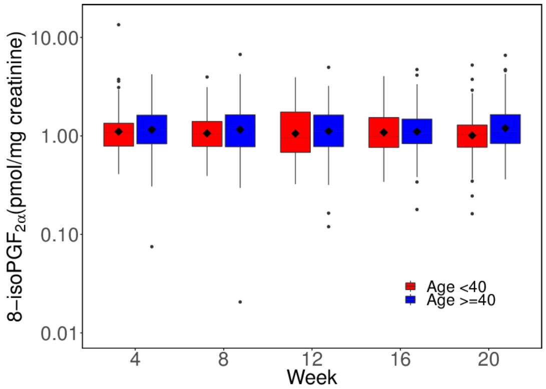

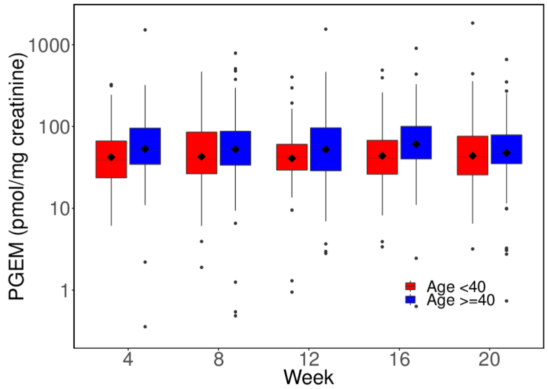

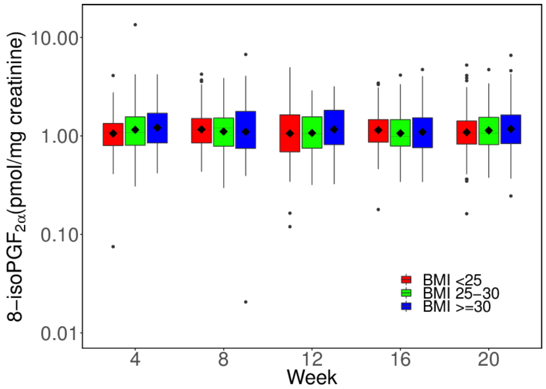

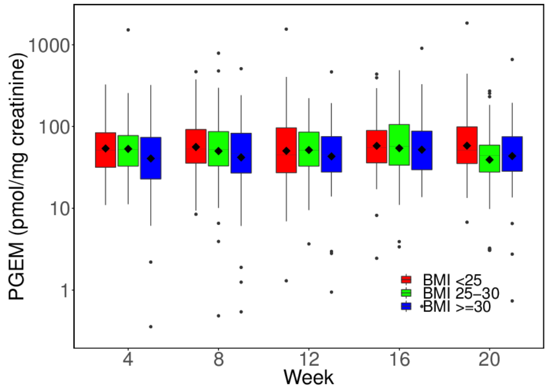

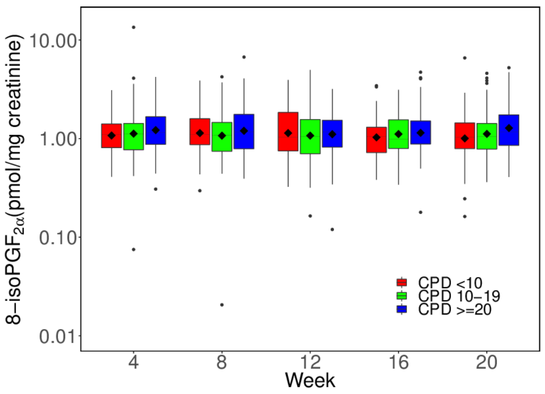

8-isoPGF2α(pmol/mg creatinine)
10.00
1.00
0.10
0.01
4
8
12
16
20
Week
CPD <10
CPD 10-19
CPD >=20

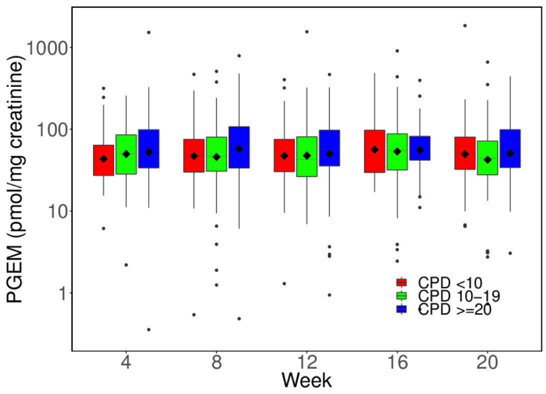

PGEM (pmol/mg creatinine)
1000
100
10
1
4
8
12
16
20
Week
CPD <10
CPD 10-19
CPD >=20
