# Supplementary material for: Longitudinal stability in cigarette smokers of urinary eicosanoid biomarkers of oxidative damage and inflammation
Source: PLoS One. 2019 Apr 25;14(4):e0215853. doi: 10.1371/journal.pone.0215853 (PMC6483352; doi:10.1371/journal.pone.0215853)
Supplement: S9 Supporting Information — (PDF) [file pone.0215853.s009.pdf]

S9 Supporting Information. Coefficients of variation by gender, age, BMI, and cigarettes per day.

Coefficient of variation (CV)

Summary of CV by groups of gender, age, BMI, and CPD

Variable	N	**Mean**	Median	25th Pctl	75th Pctl	Minimum	Maximum
**Creatinine-corrected**							
**8-isoPGF$_{2\alpha}$ /creatinine (pmol/mg)**							
Overall	*222*	***0.31***	*0.28*	*0.19*	*0.39*	*0.03*	*1.20*
Female	98	**0.33**	0.29	0.21	0.42	0.03	1.20
Male	124	**0.28**	0.26	0.17	0.36	0.06	0.76
Age<40	75	**0.31**	0.26	0.17	0.42	0.05	1.20
Age>=40	147	**0.30**	0.28	0.20	0.38	0.03	0.90
BMI<25	76	**0.32**	0.29	0.21	0.40	0.06	0.90
BMI 25-29	73	**0.30**	0.25	0.18	0.34	0.05	1.20
BMI>=30	73	**0.30**	0.28	0.19	0.40	0.03	0.76
CPD<10	51	**0.35**	0.31	0.22	0.46	0.05	0.76
CPD 10-19	107	**0.28**	0.24	0.18	0.34	0.03	1.20
CPD>=20	64	**0.32**	0.30	0.18	0.40	0.07	0.76
**PGEM/creatinine (pmol/mg)**							
Overall	*224*	***0.54***	*0.47*	*0.33*	*0.68*	*0.11*	*2.05*
Female	99	**0.60**	0.49	0.36	0.78	0.13	1.68
Male	125	**0.49**	0.46	0.32	0.59	0.11	2.05
Age<40	75	**0.51**	0.45	0.30	0.63	0.11	2.05
Age>=40	149	**0.55**	0.48	0.33	0.71	0.13	1.68
BMI<25	77	**0.54**	0.46	0.30	0.67	0.11	2.05
BMI 25-29	74	**0.53**	0.49	0.32	0.66	0.13	1.61
BMI>=30	73	**0.55**	0.47	0.36	0.70	0.13	1.44
CPD<10	52	**0.53**	0.45	0.32	0.60	0.14	2.05
CPD 10-19	108	**0.53**	0.47	0.31	0.68	0.11	1.68
CPD>=20	64	**0.57**	0.49	0.37	0.77	0.13	1.61
**Non-corrected**							
**8-isoPGF$_{2\alpha}$ (pmol/mL)**							
Overall	*222*	***0.45***	*0.43*	*0.29*	*0.59*	*0.06*	*1.27*
Female	98	**0.45**	0.41	0.28	0.60	0.06	0.86
Male	124	**0.46**	0.43	0.30	0.58	0.09	1.27
Age<40	75	**0.49**	0.45	0.28	0.63	0.06	1.23
Age>=40	147	**0.44**	0.42	0.29	0.56	0.09	1.27
BMI<25	76	**0.49**	0.45	0.32	0.61	0.09	1.23
BMI 25-29	73	**0.45**	0.41	0.26	0.61	0.10	1.27
BMI>=30	73	**0.42**	0.41	0.27	0.52	0.06	0.79
CPD<10	51	**0.47**	0.43	0.26	0.60	0.06	1.23

Variable	N	**Mean**	Median	25th Pctl	75th Pctl	Minimum	Maximum
CPD 10-19	107	**0.45**	0.42	0.32	0.61	0.09	1.18
CPD>=20	64	**0.44**	0.45	0.27	0.57	0.09	1.27
**PGEM (pmol/mL)**							
Overall	*224*	***0.65***	*0.59*	*0.45*	*0.80*	*0.07*	*1.75*
Female	99	**0.69**	0.62	0.48	0.84	0.07	1.68
Male	125	**0.61**	0.57	0.42	0.74	0.12	1.75
Age<40	75	**0.63**	0.60	0.50	0.74	0.18	1.39
Age>=40	149	**0.65**	0.59	0.42	0.83	0.07	1.75
BMI<25	77	**0.66**	0.59	0.45	0.82	0.14	1.59
BMI 25-29	74	**0.62**	0.63	0.42	0.75	0.17	1.75
BMI>=30	73	**0.65**	0.57	0.46	0.75	0.07	1.68
CPD<10	52	**0.65**	0.65	0.46	0.81	0.14	1.39
CPD 10-19	108	**0.64**	0.56	0.43	0.77	0.07	1.68
CPD>=20	64	**0.65**	0.59	0.46	0.84	0.22	1.75
**TNE-corrected**							
**8-isoPGF$_{2\alpha}$ (TNE corrected, ×10^3)**							
Overall	*222*	***0.42***	*0.33*	*0.24*	*0.51*	*0.09*	*1.93*
Female	98	**0.47**	0.37	0.26	0.57	0.10	1.93
Male	124	**0.39**	0.31	0.23	0.45	0.09	1.86
Age<40	75	**0.48**	0.34	0.24	0.59	0.09	1.93
Age>=40	147	**0.40**	0.33	0.24	0.49	0.09	1.30
BMI<25	76	**0.45**	0.37	0.26	0.52	0.10	1.93
BMI 25-29	73	**0.43**	0.31	0.23	0.51	0.12	1.86
BMI>=30	73	**0.40**	0.34	0.24	0.45	0.09	1.56
CPD<10	51	**0.56**	0.41	0.29	0.75	0.12	1.93
CPD 10-19	107	**0.41**	0.34	0.25	0.49	0.11	1.86
CPD>=20	64	**0.33**	0.28	0.20	0.43	0.09	0.97
**PGEM (TNE corrected, ×10^3)**							
Overall	*224*	***0.61***	*0.50*	*0.37*	*0.74*	*0.01*	*1.98*
Female	99	**0.70**	0.58	0.41	0.87	0.13	1.98
Male	125	**0.54**	0.46	0.36	0.65	0.01	1.95
Age<40	75	**0.63**	0.48	0.37	0.72	0.01	1.98
Age>=40	149	**0.60**	0.51	0.38	0.74	0.04	1.70
BMI<25	77	**0.62**	0.52	0.37	0.74	0.01	1.98
BMI 25-29	74	**0.60**	0.51	0.38	0.74	0.06	1.95
BMI>=30	73	**0.61**	0.49	0.38	0.76	0.04	1.70
CPD<10	52	**0.67**	0.59	0.38	0.81	0.15	1.98
CPD 10-19	108	**0.60**	0.50	0.37	0.71	0.01	1.95
CPD>=20	64	**0.58**	0.48	0.38	0.73	0.04	1.63
**Creatinine-and-TNE-corrected**							
**8-isoPGF$_{2\alpha}$ (uL/mg, creatinine and TNE corrected)**							

Variable	N	**Mean**	Median	25th Pctl	75th Pctl	Minimum	Maximum
Overall	*222*	***0.63***	*0.56*	*0.40*	*0.76*	*0.04*	*1.88*
Female	98	**0.69**	0.57	0.43	0.87	0.17	1.88
Male	124	**0.58**	0.54	0.38	0.73	0.04	1.75
Age<40	75	**0.69**	0.57	0.40	0.79	0.04	1.88
Age>=40	147	**0.60**	0.54	0.40	0.76	0.09	1.65
BMI<25	76	**0.62**	0.55	0.38	0.73	0.12	1.88
BMI 25-29	73	**0.65**	0.56	0.42	0.79	0.04	1.77
BMI>=30	73	**0.62**	0.56	0.41	0.76	0.09	1.77
CPD<10	51	**0.75**	0.60	0.43	0.91	0.09	1.88
CPD 10-19	107	**0.62**	0.54	0.39	0.76	0.12	1.65
CPD>=20	64	**0.55**	0.52	0.39	0.66	0.04	1.59
**PGEM (uL/mg, creatinine and TNE corrected)**							
Overall	*224*	***0.73***	*0.65*	*0.44*	*0.89*	*0.04*	*2.17*
Female	99	**0.84**	0.71	0.47	1.09	0.15	2.11
Male	125	**0.65**	0.60	0.40	0.80	0.04	2.17
Age<40	75	**0.77**	0.62	0.44	0.97	0.04	2.17
Age>=40	149	**0.71**	0.65	0.43	0.86	0.15	2.11
BMI<25	77	**0.72**	0.70	0.42	0.90	0.04	2.17
BMI 25-29	74	**0.75**	0.66	0.44	0.93	0.15	2.05
BMI>=30	73	**0.72**	0.60	0.46	0.86	0.15	1.91
CPD<10	52	**0.82**	0.71	0.45	1.03	0.23	2.17
CPD 10-19	108	**0.72**	0.68	0.41	0.87	0.04	2.11
CPD>=20	64	**0.67**	0.58	0.44	0.77	0.15	1.93
