# Supplementary material for: Longitudinal stability in cigarette smokers of urinary eicosanoid biomarkers of oxidative damage and inflammation
Source: PLoS One. 2019 Apr 25;14(4):e0215853. doi: 10.1371/journal.pone.0215853 (PMC6483352; doi:10.1371/journal.pone.0215853)

S10 Supporting Information. Scatter plots of CV for continuous age, BMI, and CPD.

Age

CV of 8-isoPGF$_{2α}$ /creatinine (pmol/mg), 8-isoPGF$_{2α}$ (pmol/mL), 8-isoPGF$_{2α}$ (TNE corrected, ×10^3), and 8-isoPGF$_{2α}$ (uL/mg, creatinine and TNE corrected) vs. age

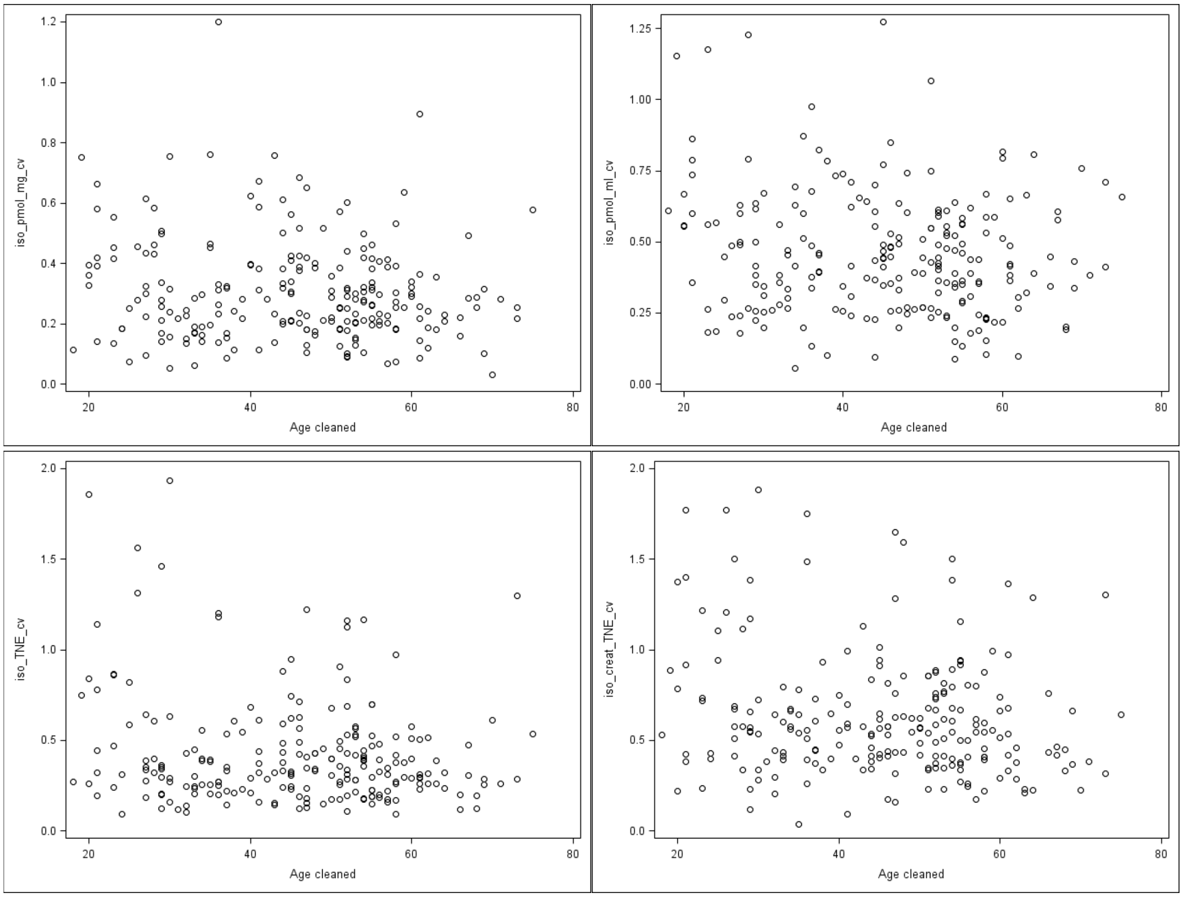

CV of PGEM/creatinine (pmol/mg), PGEM (pmol/mL), PGEM (TNE corrected, ×10^3), and PGEM (uL/mg, creatinine and TNE corrected) vs. age

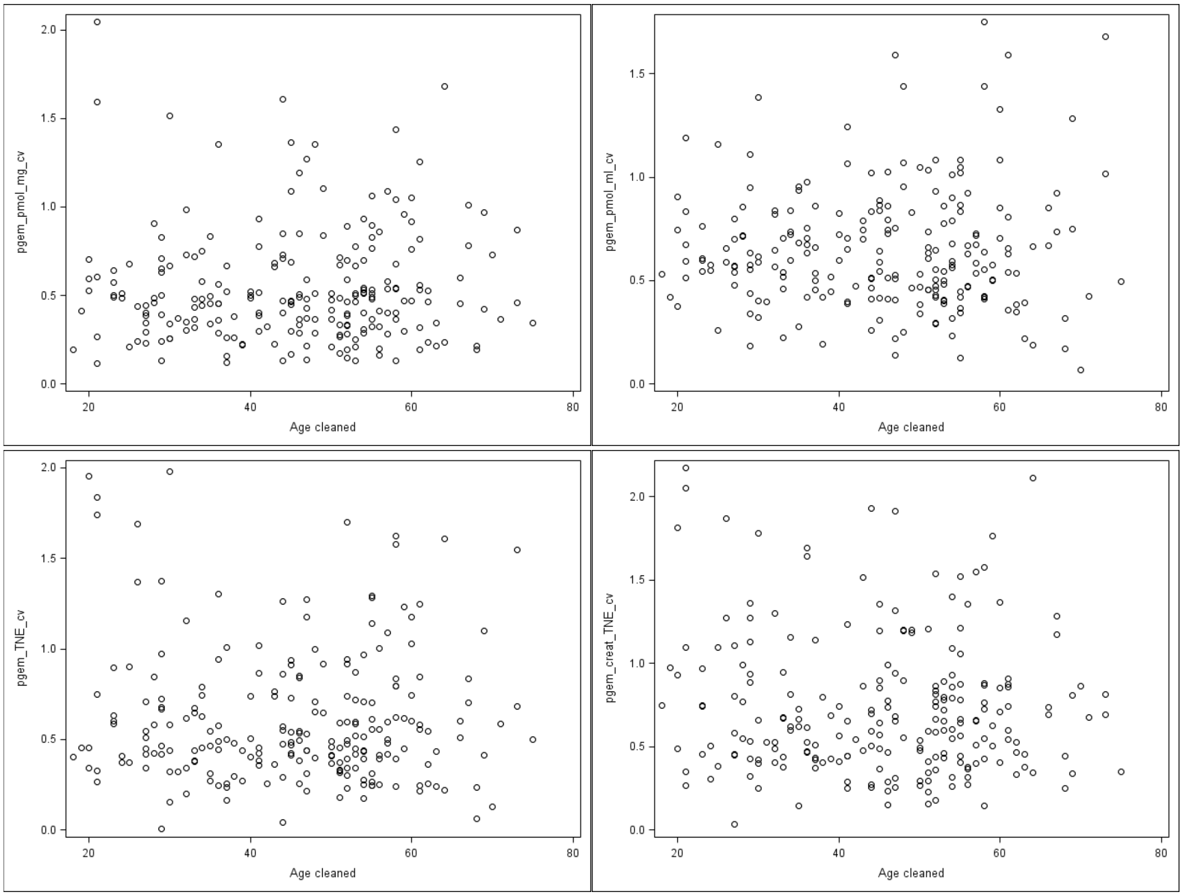

BMI

CV of 8-isoPGF$_{2\alpha}$ /creatinine (pmol/mg), 8-isoPGF$_{2\alpha}$ (pmol/mL), 8-isoPGF$_{2\alpha}$ (TNE corrected, ×10^3), and 8-isoPGF$_{2\alpha}$ (uL/mg, creatinine and TNE corrected) vs. BMI

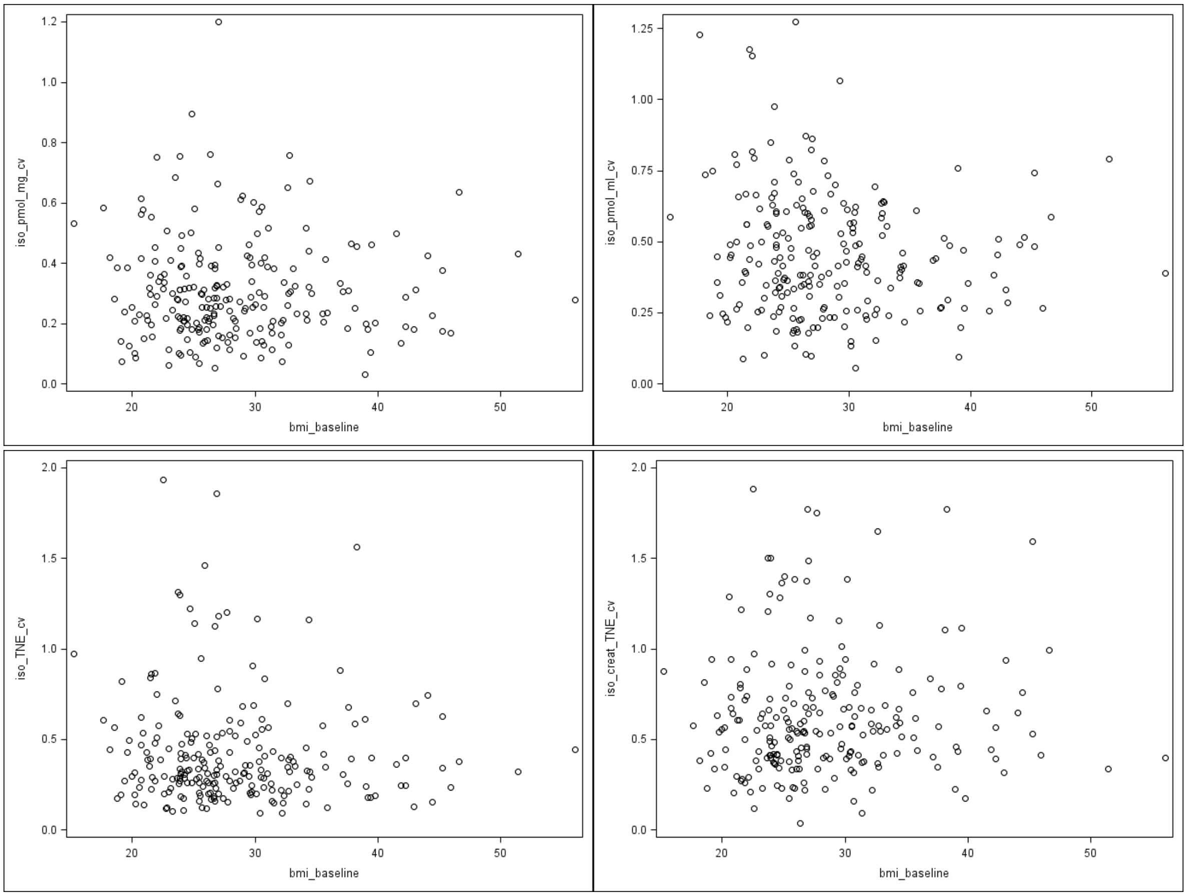

CV of PGEM/creatinine (pmol/mg), PGEM (pmol/mL), PGEM (TNE corrected, ×10^3), and PGEM (uL/mg, creatinine and TNE corrected) vs. BMI

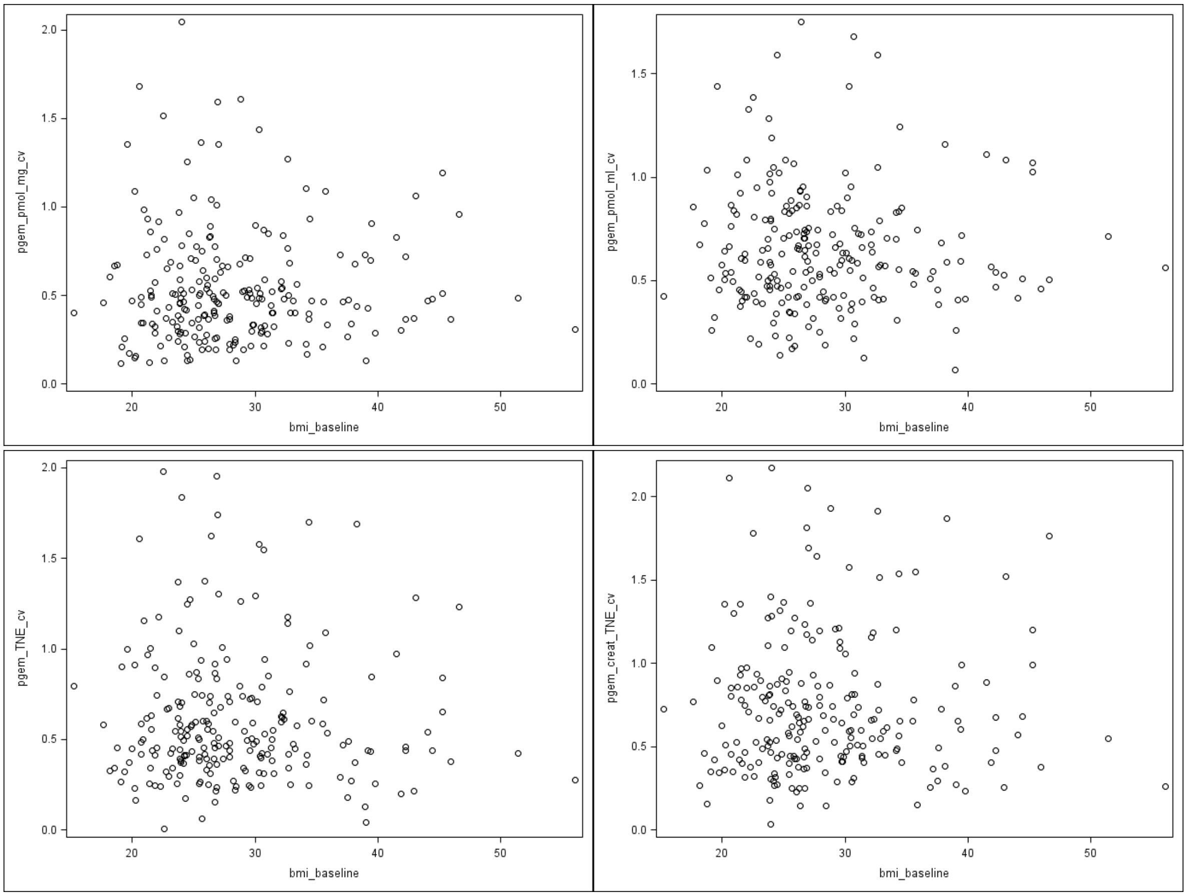

CPD

CV of 8-isoPGF$_{2\alpha}$ /creatinine (pmol/mg), 8-isoPGF$_{2\alpha}$ (pmol/mL), 8-isoPGF$_{2\alpha}$ (TNE corrected, ×10^3), and 8-isoPGF$_{2\alpha}$ (uL/mg, creatinine and TNE corrected) vs. CPD

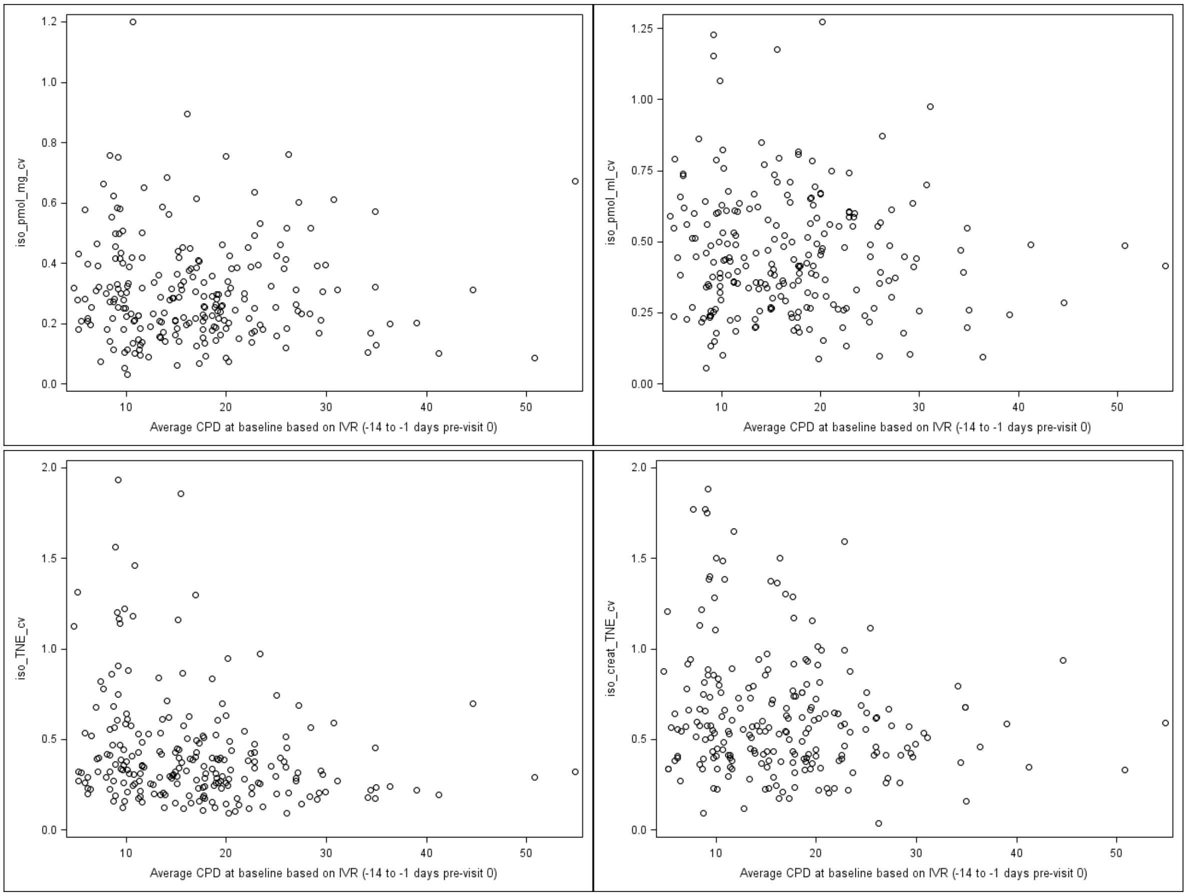

CV of PGEM/creatinine (pmol/mg), PGEM (pmol/mL), PGEM (TNE corrected, ×10^3), and PGEM (uL/mg, creatinine and TNE corrected) vs. CPD

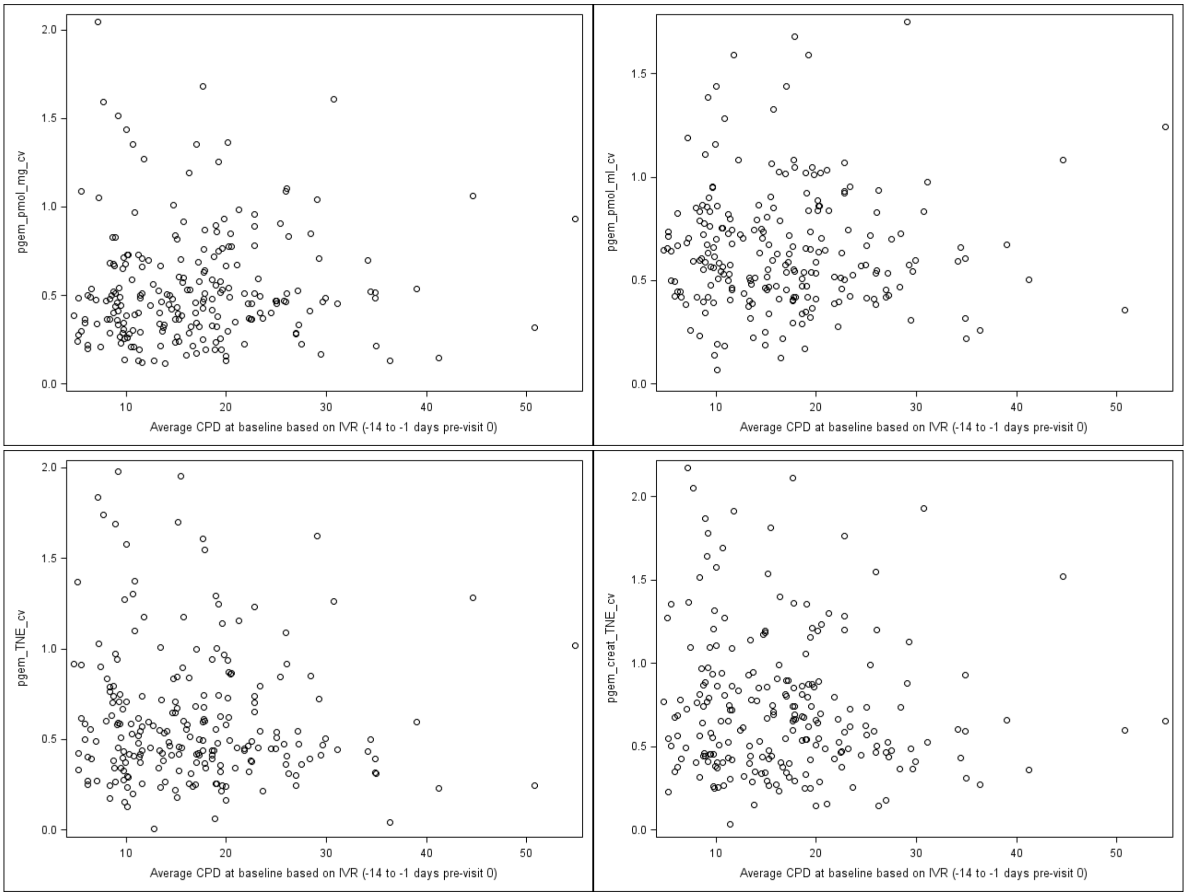

Supplement: S10 Supporting Information — (PDF) [file pone.0215853.s010.pdf]
