# Supplementary material for: Longitudinal stability in cigarette smokers of urinary eicosanoid biomarkers of oxidative damage and inflammation
Source: PLoS One. 2019 Apr 25;14(4):e0215853. doi: 10.1371/journal.pone.0215853 (PMC6483352; doi:10.1371/journal.pone.0215853)
Supplement: S11 Supporting Information — (PDF) [file pone.0215853.s011.pdf]

S11 Supporting Information. Bivariate and multivariable linear regression of CV of each biomarker on gender, age, BMI, and cigarettes per day.

Bivariate and multivariable linear regression of CV of each biomarker on gender, age, BMI, and CPD, where age, BMI, and CPD are continuous variables in the regression.

Bivariate regression p-value for each covariate, the sign in parenthesis by the significant p-value indicates the direction of the correlation

**8-isoPGF$_{2\alpha}$**	**Creatinine corrected**
**Female**	**0.0270* (+)**
**Age**	0.0661
**BMI**	0.7400
**CPD**	0.7096
**PGEM**	**Creatinine corrected**
**Female**	**0.0063* (+)**
**Age**	0.6364
**BMI**	0.9141
**CPD**	0.3949

Multivariate regression p-value of each covariate, the sign in parenthesis by the significant p-value indicates the direction of the correlation

**8-isoPGF$_{2\alpha}$**	**Creatinine corrected**	**Not corrected**	**TNE corrected**	**Creatinine and TNE corrected**
**Female**	**0.0227* (+)**	0.8557	0.0858	**0.0344* (+)**
**Age**	0.0616	0.0642	**0.0214* (-)**	**0.0161* (-)**
**BMI**	0.6380	0.1681	0.6004	0.5707
**CPD**	0.8591	0.3997	**0.0032* (-)**	0.0259
**PGEM**	**Creatinine corrected**	**Not corrected**	**TNE corrected**	**Creatinine and TNE corrected**
**Female**	**0.0044* (+)**	**0.0363* (+)**	**0.0014* (+)**	**0.0010* (+)**
**Age**	0.7766	0.7134	0.5727	0.2376
**BMI**	0.7512	0.4494	0.4916	0.9299
**CPD**	0.2350	0.5995	0.6676	0.4172

Conclusions:

- Female gender is associated with higher CV (i.e., worse longitudinal stability) for all biomarkers except for two.
- Older age is associated with smaller CV (i.e., better longitudinal stability) for TNE-corrected and creatinine-and-TNE-corrected 8-isoPGF2α, but not other biomarkers.
- Smoking more cigarettes per day is associated with smaller CV (i.e., better longitudinal stability) for only TNE-corrected 8-isoPGF2α.
