# Supplementary material for: Longitudinal stability in cigarette smokers of urinary eicosanoid biomarkers of oxidative damage and inflammation
Source: PLoS One. 2019 Apr 25;14(4):e0215853. doi: 10.1371/journal.pone.0215853 (PMC6483352; doi:10.1371/journal.pone.0215853)
Supplement: S12 Supporting Information — (PDF) [file pone.0215853.s012.pdf]

S12 Supporting Information. Related analysis for C-Reactive protein (CRP).

CRP Analysis (11/22/2018)

Note: In the JAMA paper, CRP was analyzed two different ways, using all values and using only values ≤ 10 mg/L. In this report, all CRP values were used.

Summary by week

Analysis Variable : CRP_LOD_imputed CRP (mg/L), LOD imputed with 1/2 of LOD (0.5*0.15=0.075)

visitnum	N Obs	N nonmissing	Mean	Std Dev	Std Error	Minimum	Maximum	Median	Geometric Mean
4	249	226	4.182	6.814	0.453	0.075	47.870	1.840	2.002
8	235	207	3.612	5.350	0.372	0.260	58.040	1.980	2.077
12	225	205	4.805	9.435	0.659	0.170	76.340	2.180	2.300
16	218	197	3.980	5.796	0.413	0.150	50.260	2.230	2.204
20	216	199	4.148	6.006	0.426	0.210	45.990	2.290	2.217

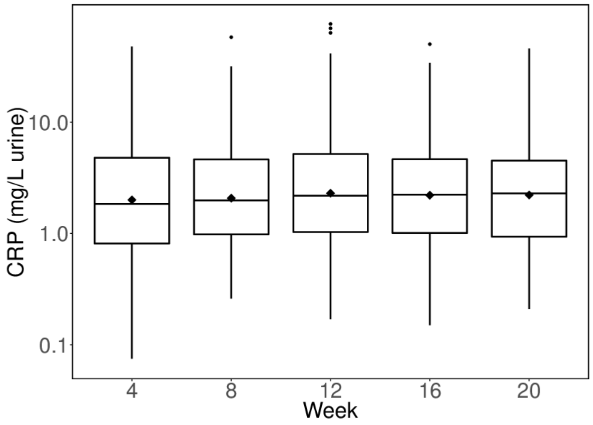

Repeated measures analysis for each covariate

Outcome Variable	Covariate	Estimated fixed effect (95% CI), p-value
Log(CRP)	Female	0.01 (-0.25, 0.27)<br>p = 0.92
	Age (×10 years)	0.19 (0.10, 0.29)<br>**p < 0.0001**
	BMI (kg/m^2)	0.08 (0.06, 0.09)<br>**p < 0.0001**
	CPD (×10)	0.01 (0.00, 0.03)<br>p = 0.06

Conclusion: Older age and larger BMI were significantly associated with higher CRP level.

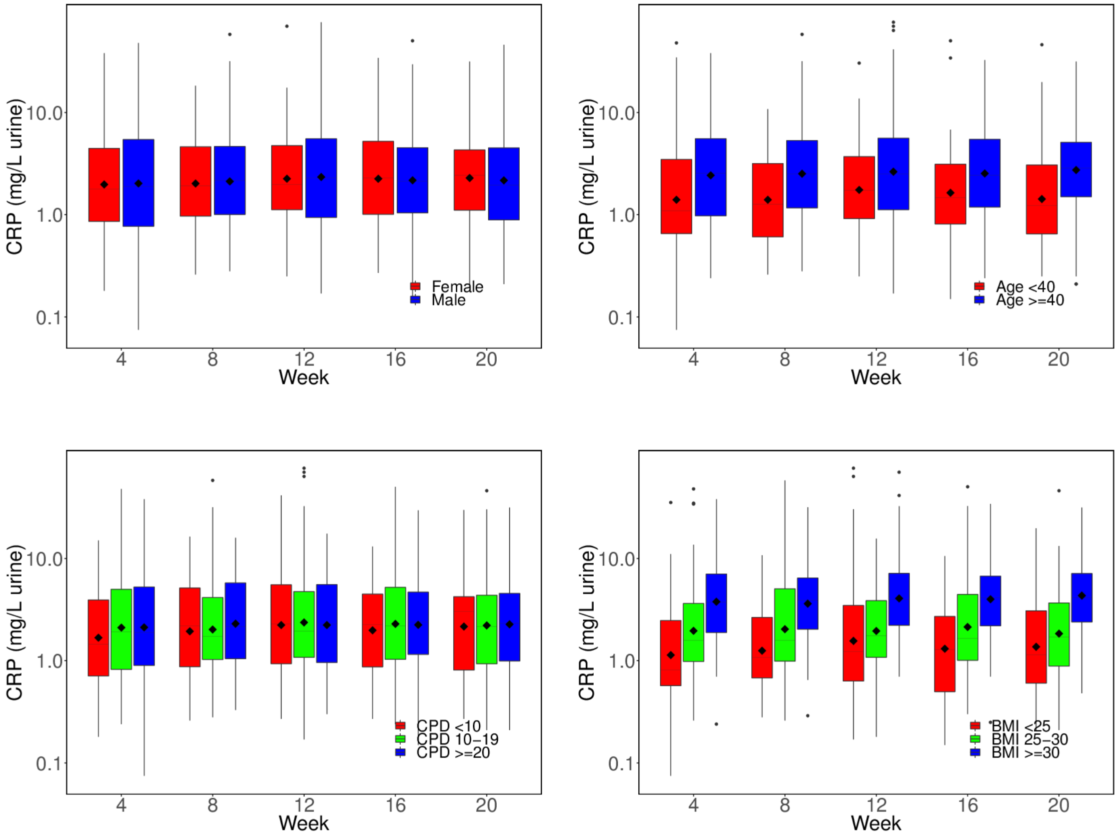

Coefficient of variation (CV)

Variable	CV						
	N	Mean	Median	25th Pctl	75th Pctl	Minimum	Maximum
Overall group	*216*	***0.50***	*0.35*	*0.23*	*0.61*	*0.04*	*2.00*
Female	94	**0.46**	0.33	0.22	0.56	0.09	1.84
Male	122	**0.54**	0.38	0.24	0.73	0.04	2.00
Age<40	73	**0.60**	0.41	0.20	0.92	0.05	2.00
Age>=40	143	**0.46**	0.34	0.24	0.55	0.04	1.82
BMI<25	76	**0.56**	0.37	0.24	0.77	0.05	1.84
BMI 25-29	70	**0.57**	0.39	0.28	0.73	0.09	2.00
BMI>=30	70	**0.37**	0.31	0.17	0.46	0.04	1.84
CPD<10	51	**0.45**	0.35	0.26	0.60	0.09	1.35
CPD 10-19	106	**0.58**	0.36	0.23	0.79	0.04	2.00
CPD>=20	59	**0.41**	0.36	0.19	0.51	0.06	1.47

Bivariate regression p-value for each covariate, the sign in parenthesis by the significant p-value indicates the direction of the correlation

Covariate	p-value (direction of correlation)
**Female**	0.2033
**Age**	0.0720
**BMI**	**0.0134 (-)**
**CPD**	0.3344

Multivariate regression p-value of each covariate, the sign in parenthesis by the significant p-value indicates the direction of the correlation

Covariate	p-value (direction of correlation)
**Female**	0.2270
**Age**	0.1047
**BMI**	**0.0251 (-)**
**CPD**	0.5406

Conclusion: Higher BMI was significantly associated with lower CV, i.e., better longitudinal stability.

ICC

Variable	Factor	Level	N of subjects	ICC (95% CI)
Log(CRP)	Overall	-	227	0.72 (0.67, 0.76)
	Gender	Female	99	0.75 (0.68, 0.81)
		Male	128	0.70 (0.64, 0.76)
	Age	<40	79	0.60 (0.50, 0.70)
		≥40	148	0.76 (0.70, 0.81)
	BMI	<25	79	0.62 (0.52, 0.71)
		25-29	74	0.63 (0.53, 0.72)
		≥30	74	0.78 (0.71, 0.84)
	CPD	<10	53	0.80 (0.72, 0.87)
		10-19	110	0.63 (0.55, 0.71)
		>=20	64	0.81 (0.74, 0.87)
